# To Facilitate or Curb? The Role of Financial Development in China’s Carbon Emissions Reduction Process: A Novel Approach

**DOI:** 10.3390/ijerph14101222

**Published:** 2017-10-13

**Authors:** Tiancai Xing, Qichuan Jiang, Xuejiao Ma

**Affiliations:** 1 School of Finance, Dongbei University of Finance and Economics, Dalian 116023, China; xingtiancai@126.com; 2 School of Statistics, Dongbei University of Finance and Economics, Dalian 116023, China; xuejiaomadufe@163.com

**Keywords:** financial development, carbon emissions, financial development index system, STIRPAT model, dynamic panel data analysis, regional and stage analysis

## Abstract

With the Paris Agreement coming into effect, China, as the largest CO_2_ emitter in the world, will be facing greater pressure to reduce its carbon emissions. This paper discusses how to solve this issue from the perspective of financial development in China. Although many studies have analyzed its impact on carbon emissions, the conclusions are contradictory. A major criticism of the existing studies is the reasonability of the selection of appropriate indicators and panel estimation techniques. Almost all studies use only one or limited indicators to represent the financial development and ignore the cross-sectional dependence. To fulfil the gaps mentioned above, a financial development index system is built, and with the framework of the STIRPAT (Stochastic impacts by regression on population, affluence, and technology) model, this paper applies an ARDL approach to investigating the long-run relationship between financial development and carbon emissions and a dynamic panel error-corrected model to capture the short-run impact. The empirical results show that financial development can improve carbon emissions, and such impact not only shows a regional difference but also reflects the features of stage differences. Additionally, based on the discussion on seven specific aspects of financial development, our findings can be helpful for policy makers to enact corresponding policies to realize the goal of reducing carbon emissions in China.

## 1. Introduction

The notion that only developed countries face environmental degradation is invalid, at least in terms of consequences, because the greenhouse gases have an enormously negative impact on both developing and developed countries regardless of the source of the gases [1]. The burning of fossil fuels and human activities results in a large increase in the concentration of greenhouse gases, which causes the extreme climate events and seriously deteriorates living conditions for humans [2]. On the one hand, global warming will aggravate destruction of the environment, which will result in a more frequent and lasting extreme climate, such as tornadoes and droughts. From 1900 to 2015, the global average temperature rose by 1.02 °C [3]. On the other hand, climate change is greatly challenging the sustainability of human society and threatening human health, causing respiratory disease and malnutrition [4]. 

**Figure 1:**
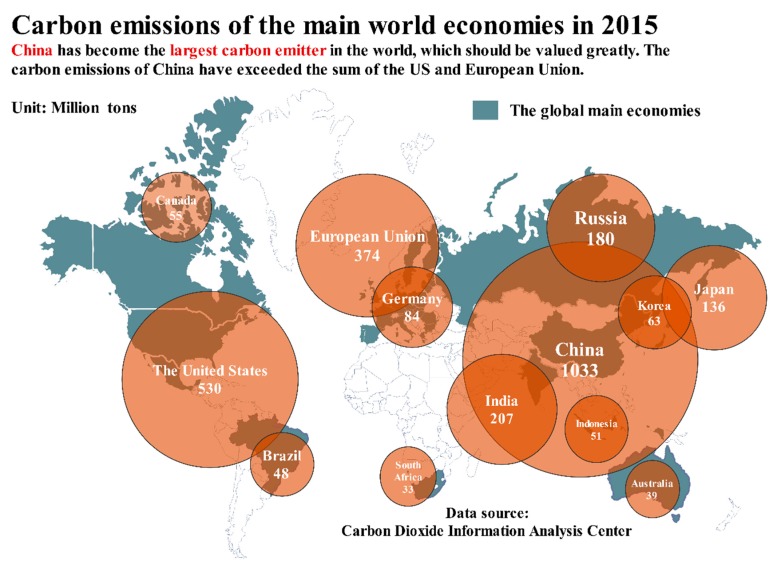
Carbon emissions of the main world economies in 2015.

**Figure 2:**
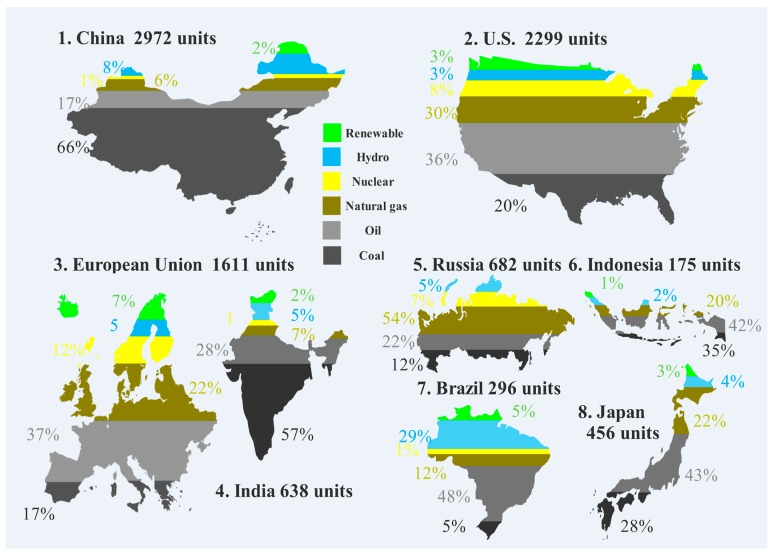
Energy structure of main economies in the world.

According to Global Carbon Projects [5], the total amount of carbon emissions and carbon emissions per capita is 36 billion tons and 5 tons, respectively, which set a new historical record. The concentration of all greenhouse gases continues to increase, and the greenhouse gas index in 2015 is 37% higher than that in 1990, among which the contribution of CO_2_ is approximately 80%. Figure 1 demonstrates the distribution of carbon emissions in the world, which also verifies that China has surpassed the European Union and the U.S. and has become the largest carbon emitter in the world [6]. In Figure 2, the energy structure of China, the U.S., the European Union, India, Russia, Indonesia, Brazil and Japan can be seen clearly. Coal, oil and natural gas are three main sources for carbon emissions. 

To address the issue of climate change, the global community has agreed that each country is to adopt measures to alleviate global warming, such as improving energy use efficiency [7]. The European Union has implemented an *Emission Trading Scheme* and proposed to reduce carbon emissions by approximately 20–30% by 2020. The UK was the first county to include emission reduction into a legal framework. China began to focus on energy and environmental problems in the 21st century, and a series of effective measures have been taken. In 2009, China promised that carbon emissions reduction would be included in the middle- and long-term development plan for the national economy and social development as an obligatory target, and the carbon emission intensity (CEI) in 2030 would decrease by 60–65% compared to that in 2005 [8], which shows a great determination on China’s part to reduce carbon emissions. Since 2000, China has made large contributions to reducing carbon emissions, and its detailed policies are listed in Appendix A, Table A1.

According to CDIAC [9], the Chinese carbon emissions levels have staged characteristics, which can be divided into the following four phases: (1) 1978–1995. The emission of carbon dioxide increased year by year with a relatively slow growth rate of approximately 5.3%; (2) 1996–2001. Carbon emissions nearly stopped and decreased; (3) 2002–2009. China’s carbon emissions increased rapidly, and in 2006, China surpassed the U.S., becoming the largest carbon emitter in the world; (4) 2003–2015. Growth again slowed. 

During the Asian financial crisis in 1997, the Chinese financial development had been in a downward state when the deflation occurred. After 2002, China began to decrease the deflation and growth rate of both carbon emissions and GDP. In 2008, the American subprime mortgage crisis triggered a global financial crisis, which pushed the Chinese finance into a brief recession. Therefore, at the end of 2008, China implemented a simple monetary policy and proactive fiscal policy to promote the rapid growth of energy consumption and financial development. Then the broad money supply M2 in 2013 was 11.07 billion yuan, which occupies a leading position in the world. Carbon emissions and financial development peaked from a global perspective almost simultaneously [10]. Based on the rules above, there is a high correlation between financial development and carbon emissions in China. Therefore, the influences of financial development on carbon emissions should be researched in depth. 

Financial development may both stimulate the increase of carbon emissions and promote its reduction [11]. On the one hand, financial development has both a wealth and scale effects [12]. For the wealth effect, the prosperity of the financial market can allow customers to obtain wealth and capital more conveniently, which would satisfy the needs of the customers for energy consumption products and encourage them to purchase more cars, houses and so on, which would obviously increase carbon emissions. Meanwhile, the expansion of financial development and the capital market is beneficial for the expansion of the production scale of enterprises and marketing activities, which encourages the adoption of financing to build new production lines and purchase large-scale equipment to expand production. Thus, the scale effect of financial development on carbon emissions is clear.

However, financial development can also have a technological and structural effect on carbon emissions [12] because the financial development and prosperity of a capital market can attract more foreign direct investment with high technology and more investment for research and development, promoting technological advancement and curbing carbon emissions in local regions. At the same time, a developed financial market prefers investment in environmentally friendly projects, which can offer more convenient financing and motivation for new projects and facilities that have the advantages of energy conservation and emission reduction or market potential. Therefore, the industry and energy structure can be enhanced, and the structural effect of financial development on carbon emissions is apparent, which could promote the development of a low-carbon economy.

Based on the discussion above, in theory, the relationship between financial development and carbon emissions can be positive or negative; thus, the practical correlation needs to be analyzed more deeply and thoroughly. This paper proposes a novel method to solve this issue, which combines the establishment of an index system for financial development, the application of an extended STIRPAT model and the use of an error correction-based model based on cross-sectional independence. The main practical and theoretical significance of this paper is listed as follows:

(1) With environmental degradation and resource exhaustion, all countries face great pressure regarding carbon emissions. In total, 175 countries have signed the Paris Agreement, which is a positive and solid step towards jointly face one of the most important long-term challenges currently: climate change. As the largest carbon emitter, China’s efforts to reduce carbon emissions can notably contribute to mitigating global warming. Therefore, researching carbon emissions in China is of great significance. Additionally, the influences of many factors on carbon emissions, such as urbanization and population, have been clear. However, there are mixed conclusions on how financial development affects carbon emissions due to different research approaches, selected variables and so on. In fact, with the integration of the global economy and the rapid development of the financial industry, financial development has played an increasingly pivotal role in influencing carbon emissions. Thus, this research has great practical significance.
(2) The approach of this paper in researching the relationship between carbon emissions and financial development is different from previous studies in the following aspects:
  ◆ When measuring financial development, we creatively establish a comprehensive evaluation index system to represent it instead of a single variable. To the best of our knowledge, it is impossible to fully measure financial development by using only one index, which will have an influence on the reliability of the analysis results. Additionally, giving proper weight to each individual variable in the index system plays a pivotal role in deciding the effectiveness of the system. Therefore, this paper combines a sequential global principal component analysis and the entropy weight method to ensure that the correct weight is used, which can overcome subjectivity and leverage each method fully.
  ◆ When calculating carbon emissions, this paper considers both the combustion of 13 types of fossil fuels and the production of cement, which can make the calculation of carbon emissions more accurate and comprehensive.
  ◆ The ARDL approach is applied to investigate the long-run relationship between financial development and carbon emissions, and a dynamic panel error-corrected model is utilized to capture the short-run impact. Before estimation, we test whether each region is independent, after which a cross-section mean group (CMG), pooled mean group (CPMG) and dynamic fixed effect (CDFE) are applied to estimate the parameters in the equation.
  ◆ The fourth feature of the proposed method is the application of the STIRPAT model. It has been proven to be effective in researching the influencing factors of carbon emissions, and one apparent advantage is that the model can be expanded to include the financial development index, apart from some common factors.
  ◆ Apart from discussing the overall impact, we also divide China into different regions and stages to explore the regional and stage differences of the impact. Different from the time series data, the panel data can be more representative and effective in researching Chinese carbon emissions. Furthermore, we also discuss the impact of seven specific aspects of the financial development index system on the carbon emissions level.
(3) Our research results not only provide the basis for policy makers in China to control carbon emissions but can also explain the relationship between financial development and carbon emissions in other developing countries in the world, such as India. Currently, financial development can facilitate carbon emissions, indicating that the wealth and scale effects are larger than the technology and structure effects. Moreover, the relationship is different in different regions and stages. With the extensive economic growth mode, financial development can lead to the increase of the carbon emissions level, and financial development will gradually show a tendency to constrain the carbon emissions level as the policies and economic structure change.

The remainder of this paper is arranged as follows: Section 2 presents a literature review. The methodology is described in Section 3, and Section 4 introduces the data and variables. Section 5 shows the empirical results. Section 6 discusses the conclusions and policy recommendations. 

## 2. Literature Review

### 2.1. Methods to Measure the Influencing Factors of Carbon Emissions

Currently, main types of methods that are applied to measure the influencing factors of carbon emissions include: the factor decomposition analysis, STIRPAT model, and models based on an environmental Kuznets curve (EKC). The index decomposing analysis (IDA) is a commonly used method belonging to the factor decomposition analysis category that studies the driving factors of carbon emissions. The IDA model is inherited from the IPAT model with the index number concept for the decomposition analysis. The model needs less data and can be applied to study spatial and time series data. Several types of IDA models are available, such as the most well-known Kaya identity and the most generally applied Laspeyers index and Divisia index [13,14,15,16,17]. However, sectors are highly aggregated when using IDA models, which can limit policy decisions to the sectoral or product scale. Some key factors are usually ignored when using IDA models due to the limitation of data sources and model framework. Therefore, many scholars [18,19,20,21] choose to use the STIRPAT model to research the influencing factors of carbon emissions because the STIRPAT model can be expanded to incorporate additional factors; as a result, it has become well known for uncovering the impact factors of pollutant emissions [22]. The STIRPAT model is flexible for testing certain hypotheses, such as whether elasticities differ across development levels, whether a population’s elasticity is different from unity and so on. If the elasticity of the population is one, the variable of population is often removed via division, so the dependent variable would be in a per capita form, and the EKC framework usually applies this form [23,24]. The essence of EKC is to determine whether there is an inverted-U shape between the economy and environment. When the environmental variable is carbon emissions per capita, EKC refers to the carbon Kuznets curves (CKC) [25,26,27,28]. Both EKC and CKC assume that pollution will show a rising trend first and then present a decreasing tendency after a certain threshold level of income is reached [29,30]. The CKC model is combined with energy-GDP causality by adding energy consumption as an explanatory variable to the classic CKC model. The new method is called the emission-energy-output (EEO) model [31,32,33,34]. Table 1 summarize the abovementioned literature. 

### 2.2. Financial Development Indexes 

The level and quality of financial development can be measured by indexes of financial development. From the appearance of the financial interrelation ratio (FIR) to the current various financial development indexes, the function and connotation of financial development have been reflected more comprehensively and clearly. Goldsmith [35] creatively developed FIR to measure the financial development level in a country or region, and it is the ratio of the market value of all financial instruments to the market value of national wealth. However, FIR is simplified, using GDP as the denominator and the total value of financial assets as the numerator. The appearance of FIR laid a foundation for follow-up studies of financial development.

Mckinnon [36] proposed the well-known theory of financial depth and indicated the basic indexes to measure financial depth, which is the ratio of M2 to GDP. However, the above two indexes are not appropriate for application in China owing to their limitations.

In the 1990s, new indexes were developed to represent financial development. King and Levine [37] presented Private and Privy indexes. The Private index was the ratio of credit and loans of the non-financial private sector to the total quantity of credits and loans. The Privy index was the ratio of credits and loans of the non-financial private sector to GDP. The increase of the two indexes can reflect the improvement of the efficiency of finance resource distribution. Levine and Zervos [38] developed a type of index to measure whether the stock market was active to reflect the financial development level. The method included three indexes: the capitalization rate, turnover rate and trading rate. The capitalization rate was the ratio of the total market value of stock in the stock exchange to GDP, the turnover rate was the ratio of trading volume in the domestic stock market to total market value of stock in the stock exchange, and the trading rate was the ratio of trading volume in the domestic stock market to GDP. 

Next, Khan and Senhadji [39] established four simplified financial indexes based on previous studies and the *International Financial Development Statistics*, which are the ratio of domestic credit in non-official departments to GDP, the ratio of the sum of domestic credit in non-official departments and total market value in the stock market to GDP, the ratio of the sum of total market value in the stock market and total market value of debt in the public sector to GDP, and the ratio of the market value of stock to GDP. Additionally, Beck and Levine [40] proposed an index system to reflect the overall level of financial development and financial structure. The former included financial liveliness, financial scale, financial size and dummy variables, and the latter included structural liveness, structural scale, structural size, structural dummy variables and structural regulation. 

Afterward, a large number of empirical studies have been conducted, but no pioneering indexes were proposed to measure financial development. In general, as shown in Table 2, indicators can be divided into three types. The ratio of real domestic credit to private sector per capita is an indicator that would be taken into consideration by most scholars. Foreign direct investment is also an important factor to measure financial development; however, its impact on carbon emissions is not unified. Additionally, indicators that measure the development of the stock market are also applied to reflect financial development more comprehensively. In current studies on the relationship between financial development and carbon emissions, most scholars use only one indicator to represent the overall situations of financial development, which is one-sided and lacks representativeness. Moreover, the problem of how to ensure the weight of each index is also not solved; thus, they fail to reflect the real level of financial development. In addition, simply applying the above indexes to study the impact of financial development on the carbon emissions in developing countries has major limitations. Therefore, this paper redefines the connotation of financial development and builds a scientific, systematic and comprehensive evaluation index system to represent financial development. 

### 2.3. Methods to Research the Relationship between Carbon Emissions and Financial Development

A large variety of methods are applied to study the relationship between carbon emissions and financial development, including the ordinary least square-OLS [41], Johansen panel cointegration and vector error correction-based Granger causality [42,43], autoregressive distributed lag-based cointegration and Granger causality [44,45,46,47,48], static panel data analysis [49], dynamic panel data analysis [50,51] and heterogeneous panel data analysis [52]. A number of studies focus on cross-regional research with multiple regions and a long time period among the studies reviewed, so the dynamic panel data analysis developed by Pesaran [53] is adopted to manage such cases. 

However, from the literature, it is clear that the largest challenge for the dynamic panel data model is how to select a suitable estimating technique. In general regression models, the lagged item of the dependent variable can cause an endogeneity problem, so the conventional least square regression with fixed effect or random effect will lead to bias and inconsistency of estimated coefficients. To handle this issue effectively and successfully, the generalized method of moment (GMM) based on difference was developed to obtain more effective estimated results. However, when estimating the regression coefficients, it is assumed that all coefficients are homogeneous and that only the constant could vary. Both methods obtain a biased estimated coefficient. In comparison, when the panel data contain a large amount of cross-sectional and time series data, the mean group (MG), pooled mean group (PMG) and dynamic fixed effect (DFE) estimated method can be adopted. The MG method allows for all coefficients and variance of errors to be heterogeneous; however, this method is easily affected by the individual abnormal value, resulting in a biased estimation. Given that, PMG was proposed to limit the fact that the long-term coefficient in the model is homogeneous and allows heterogeneous short-term coefficients and variance of errors. Compared to MG, PMG improves the efficiency of the estimation. Additionally, DFE assumes that both long-term and short-term coefficients can be homogeneous, but the intercept item can be different. The Hausman test can be adopted to further ensure which method is more suitable and whether the long-term coefficients are homogeneous [54].

### 2.4. Nexus between Financial Development and Carbon Emissions

The financial development covers a wide range of meanings, including financial depth, financial size, financial efficiency, financial openness, financial structure, financial growth and financial ecology, highly correlating with the degree of capital flows in financial institutions, capital markets, and foreign direct investment (FDI) [55]. Zhang [56] considered that the carbon emissions in a country did not necessarily depend on its income level alone; the financial development, especially for an economic entity with ever-deepening financial systems, such as China, might be another source, and the carbon emissions could be affected by upper mechanisms. To the best of our knowledge, carbon emissions are a function of the level of consumption/wealth, the energy intensity of that consumption, the energy intensity of the production of goods and services, and the carbon intensity of energy. Therefore, it is necessary to discuss how various financial measures affect those four aspects. 

First, the financial development can have a direct influence on consumption level through providing residents with consumption credit services and indirectly affect the tendency and demand of their consumption. With the continuous optimization of financial structure, the financial risks become lower, so customers can plays a more positive role in the financial activities, such as investment and finance [57]. The consumption credit services and other financial products can effectively relieve liquidity constraint of income groups, improving the consumption level [58,59,60]. Additionally, according to the Keynes consumption function, the increase of capitals can also improve the level of consumption, and the financial institutions can change the total utility of residents through providing consumption credit services. Furthermore, financial institutions can further stimulate the consumption level through offering the support of technology and capitals to improve the ability of bearing risks and improve liquidity constraints.

Second, for consumers, on the one hand, when the financial development is improved, consumers can borrow more easily in order to purchase “large-ticket” items, thereby increasing the consumption in energy (when such items include automobiles, air conditioners and machinery) [61,62]. On the other hand, the constantly perfected institutional system and service system of finance can facilitate the green credit and research and development of low-carbon financial product innovation [63]. With the continuous optimization of financial ecology, the establishment of low-carbon products certification system and support of low-carbon products development can provide more detailed channels for carbon emissions reduction. During the progress of financial depth, the confidence of consumers and business is built, which can lead to expansion in the economy and create demand for energy intensive products [64]. 

Third, for companies, the development and perfect of financial system can attract more foreign investment, which could transform idle capitals into working capitals, accelerate the capital formation and accumulate creative talents (financial size); the perfected financial system can also create more financial tools and products and increase the scale of credits and securities market to reduce information asymmetry, increase corporate financing channels and optimize enterprises’ liability structure (financial growth); the financial development can improve the investment efficiency though improving efficiency of resource allocation (financial efficiency). In a nutshell, companies can easier get access to capitals in lower costs to expand the production, which can increase the energy demand. On the contrary, the financial development can also provide capital support to encourage more companies to apply newer energy-efficient equipment to decrease energy demand. Many financial sectors can play a significant role in energy transitions. For example, the banking sector has been the main source of external finance for energy investments in most countries, with capital markets offering another alternative [65]. Capital-intensive energy production of all types can benefit from larger supplies of financial capital. When there is a larger supply of aggregate financial capital available, greater competition between capital providers helps to lower the cost of capital. If the financial capital is insufficient, some energy projects may no longer be commercially viable, due to the elevated cost of capital [66]. 

Finally, the carbon intensity of energy can reflect the degree of carbon-reduction technology in a region. It is supposed to expand financial openness, accelerate financial depth and improve financial efficiency to promote the technological progress, and further reduce carbon emissions. According to the analysis above, it can be known that the financial development can affect the carbon emissions through influencing the energy consumption of production and consumption, and such impact can be positive or negative. On the one hand, financial development can promote financial openness, which means that trade between nations becomes more frequent [67]. For developing countries, the rapid development of foreign trade will boost the domestic economic growth. However, most of the export products have high energy consumption and low additional value; that is, the increase of trade is at the expense of the environmental pollution. According to the pollution haven hypothesis, developed countries will regard the developing countries as the pollution haven [68]. On the other hand, the trade of new high-tech products and the service trade control can obviously lead to the carbon emissions reduction. 

In general, whether the financial development increases or decreases the energy consumption depends on the sum of each effect. As shown in Table 2, the literature shows mixed results, which can be summarized as follows: Shahbaz et al. [45] and Charfeddine and Khediri [2] both proved the inverted-U shaped relationship between financial development and carbon emissions. Financial development can play a positive and significant role in combating environmental degradation in a country [69], and Jalil and Feridun [70] argued that financial development could lead to a decrease in environmental pollution. Salahuddin et al. [71], Mulali and Sab [72], Mulali and Sab [73] and Dogan and Seker [74] all supported the view that financial development was found to reduce CO_2_ emissions, and CO_2_ emissions had an impact and a positive causal relationship with financial development in the short and long run. However, Ozturk and Acaravci [75] and Abbasi and Riaz [76] held a different view, in which the financial development variable had no significant effect on per capita carbon emissions in the long run and financial development had led to the increase of CO_2_ emissions. Gokmenoglu et al. [77] and Boutabba [78] proved a unidirectional causality relationship between carbon emissions and financial development. Other research results focus on the impact of different indicators, such as financial openness [1,79], FDI [56,80] and the credit and stock market [11]. 

The mixed nature of the empirical findings investigated above can primarily be attributed to the individual researcher’s selection of methods, research periods and regions, and variables [81]. Although the studies above are able to enrich our understanding of the impacts of financial development of the carbon emissions level, they have failed to offer reasonable and sufficient evidence on the inner relationship between the two aspects, a significant factor for the government in developing supporting policies for financial development and carbon emissions reduction. In addition, although scholars have conducted a considerable number of studies on the relationship between carbon emissions and financial development, they only use one or several indicators to represent financial development, which is one-sided and cannot reflect the real context of Chinese financial development.

Furthermore, to simply apply these indicators to an analysis of China has great defects because they cannot represent the financial development of China properly. Therefore, this paper constructs a comprehensive index system of financial development that is in accordance with the actual Chinese context to explore its impact on carbon emissions. In addition, we also divide the comprehensive index into seven sub-aspects to study their relationship with carbon emissions. Additionally, few studies have focused on the error-correction mechanism when analyzing the long-term equilibrium relationship, so this paper applies an error correction-based dynamic panel data model to analyze the influences to overcome this issue. Furthermore, the financial development of China in different regions and stages are different; therefore, it is necessary to analyze the influences of regional financial development on carbon emissions to propose regional financial support policies to reduce carbon emissions. Thus, the research of this paper is of great innovation significance.

## 3. Methodology

### 3.1. Sequential Global Principal Component Analysis (SGPCA)

Essentially, PCA is a variant of a multivariate analysis that is based on the data analytic technique and attempts to reveal the multivariate structure of the data [82]. The purpose of this paper is to construct a new composite indicator, the financial development index (FDIN). The use of a multivariate method, such as PCA, is necessary to manage the simultaneous treatment of a series of variables [83]. PCA is performed by identifying the Eigen structure of the correlation matrix of the original data [84]. 

Assume that the original variables are $X_{1},X_{2},\ldots ,X_{n}$, and the main steps of PCA are as follows:

Step 1 *Z*-score normalization. The first step for processing the original data is to eliminate the dimension of data. The equation is
  (1) $$X_{ij}^{\prime }=(X_{ij}-{\bar{X}}_{i})/\sigma_{i},i=1,2,\ldots ,n;j=1,2\ldots ,P$$
  where $X_{ij}$ is the original data of the *i*th index and *j*th sample. ${\bar{X}}_{i}$ and $\sigma_{i}$ represent the mean and standard deviation of *i*th index, respectively.
Step 2 According to the normalized data matrix ${\left(X_{ij}^{\prime }\right)}_{n\times p}$, calculate the correlation coefficient matrix $R={\left(r_{ij}\right)}_{n\times n}$.
Step 3 Calculate the eigenvalue and eigenvector of *R*. The eigenvalue is calculated based on the characteristic equation |R−λI|=0, which is the eigen-polynomial of $r_{n}\lambda^{p}+r_{n-1}\lambda^{p-1}+\ldots r_{1}\lambda +r_{0}=0$. Solve and order $\lambda_{1},\lambda_{2},\ldots ,\lambda_{p}$, ranging in size:
  (2) $$\lambda_{1}\geq \lambda_{2}\geq \ldots \lambda_{p}\geq 0$$

List the eigenvector with regard to the eigenvalue $\lambda_{k}$.

(3) $$L_{k}={\left[L_{k1},L_{k2},\ldots ,L_{kp}\right]}^{T},RL_{k}=\lambda L_{k}$$

In general, when there are many variables, the Jacobi method is applied to calculate the eigenvalue and eigenvector.

Step 4 Calculate the contribution rate. The equation of the contribution rate and cumulative contribution rate is described in Equations (4) and (5) here:
  (4) $$\lambda_{k}/\sum_{i=1}^{p}\lambda_{i}$$
  (5) $$\sum_{j=1}^{k}(\lambda_{j}/\sum_{i=1}^{p}\lambda_{i})$$

The principal component can be extracted when the cumulative contribution rate of eigenvalues $\lambda_{1},\lambda_{2},\ldots \lambda_{m}(m\leq p)$ is above 80%.

Step 5 Calculate the principal component load matrix, which is the correlation coefficient between the principal component $Z_{k}$ and the variable $X_{i}$.
  (6) $$p(Z_{k})=\sqrt{\lambda_{k}L_{k}}(i=1,2,\ldots p,k=1,2,\ldots ,m)$$
Step 6 Scores of the principal component can be obtained with the following equations.
  (7) $$\left\{ \begin{array}{l} Z_{1}=L_{11}x_{1}^{\prime }+L_{12}x_{2}^{\prime }+\ldots +L_{1p}x_{p}^{\prime }\\ Z_{2}=L_{21}x_{2}^{\prime }+L_{22}x_{2}^{\prime }+\ldots +L_{2p}x_{p}^{\prime }\\ \ldots \\ Z_{m}=L_{m1}x_{m}^{\prime }+L_{m2}x_{2}^{\prime }+\ldots +L_{mp}x_{p}^{\prime } \end{array}\right.$$

Based on the idea of classical PCA, this paper applies SGPCA to address the panel data. The original data are ordered according to year *T* and region *S*, forming a T×S matrix. 

### 3.2. Entropy Weight Method (EWM)

The entropy-weight method is based on Shannon entropy, originally developed by Shannon [85], which is proposed as a measure of uncertainty in information and is formulated in terms of probability theory [86]. Hence, the weights identified by entropy are also the measurement of the disorder degree of the evaluation system [87]. Entropy weight represents useful information of the evaluation index [88] and is a method that can objectively determine the weights of criteria. This method is applied in many industries, such as transport systems [89], environmental time series data [90] and so on. The specific steps of EWM are described as follows:

Step 1 Non-negative data processing. Equation (8) demonstrates that entropy is a type of calculation between the individual indicator $x_{j}$ and its corresponding $X_{ij}$, which is not related to the calculation between $x_{i}$ and $x_{j}$. Therefore, there are no dimensional differences among the data, but the procedure requires non-negativity. The data processing of the large indicator and small indicator is listed in Equations (8) and (9), respectively:
  (8) $$X_{ij}^{\prime }=\frac{X_{ij}-\min(X_{1j},X_{2j},\ldots ,X_{nj})}{\max(X_{1j},X_{2j},\ldots ,X_{nj})-\min(X_{1j},X_{2j},\ldots ,X_{nj})}+1,i=1,2,\ldots ,n;j=1,2,\ldots ,m$$
  (9) $$X_{ij}^{\prime }=\frac{\max(X_{1j},X_{2j},\ldots ,X_{nj})-X_{ij}}{\max(X_{1j},X_{2j},\ldots ,X_{nj})-\min(X_{1j},X_{2j},\ldots ,X_{nj})}+1,i=1,2,\ldots ,n;j=1,2,\ldots ,m$$
Step 2 The proportion of the *j*th indicator of the *i*th scheme is
  (10) $$f_{ij}=\frac{X_{ij}}{\sum_{i=1}^{n}X_{ij}}(j=1,2,\ldots m)$$
Step 3 The entropy of the *j*th indicator is:
  (11) $$e_{j}=-k*\sum_{i=1}^{n}f_{ij}\ln(f_{ij})$$
  where k>0, ln is the natural logarithm, $e_{j}\geq 0$. The constant *k* is relative to the sample size *m*, and in general, k=1/lnn and 0≤e≤1.
Step 4 Calculate the difference coefficient of the *j*th indicator. For the *j*th indicator, a larger difference $g_{i}$ of indicator $X_{ij}$ plays a larger role in evaluating the case:
  (12) $$g_{i}=1-e_{j}$$
Step 5 The entropy weight of the *j*th indicator is:
  (13) $$W_{j}=\frac{g_{j}}{\sum_{j=1}^{m}g_{j}},j=1,2\ldots m$$

### 3.3. Combination of SGPLA and EWM

SGPCA can use a linearly independent principal component to replace the original data structure, and the weight of the principal component can be calculated by the contribution rate of factors, which effectively avoids the influence of redundant data on the evaluation results. However, the weight of indicators is concentrated, which will conversely affect the accuracy of the results. In comparison, the single EWM also has its own shortcomings. The evaluation results sometimes disregard the importance of the indicators themselves, and the weight is notably different from the expected results. Therefore, to address the issues above, this paper combines SGPCA with EWM to adjust the weight of the indicators, making the results more objective and reliable. This successfully prevents the influences of subjective factors but can also overcome the drawbacks of single objective evaluation method. The comprehensive weight model is as follows:

(14) $$\alpha_{j}=\left(W_{j}+w_{j}\right)/2$$

where $W_{j}$ is the weight obtained by SGPCA and $w_{j}$ is the weight calculated by EWM. 

### 3.4. The Expanded STIRPAT Model

The IPAT model is proposed to specify the driving forces that influence environmental pressure. In the IPAT model, (*I*) represents the environmental impacts, which is the product of three demographic and economic factors as follows: population (*P*), affluence (*A*), and technology (*T*). The IPAT model is a concise and simple way to examine the impact factors of environmental pressure, with the basic form listed in Equation (15):

(15) I=P×A×T

Using this model as a basis, the STIRPAT model is developed. The relationship between *I*, *P*, *A* and *T* is maintained in the STIRPAT model; however, it modifies some weaknesses of the IPAT model. To be specific, it rejects the unit elasticity assumption and adds randomness for convenience in the empirical analysis. Moreover, the STIRPAT model can also be used to examine several factors, such as urbanization, industrial structure and energy structure by decomposing the population and technology terms. Due to the feasibility of the STIRPAT model, this paper utilizes the model to analyse the influence of financial development on carbon emissions by using the form of expanded STIRPAT to examine the short-term and long-term relationship. The basic form of STIRPAT is listed in Equation (16):

(16) $$C_{it}=\alpha \times P_{it}^{a}\times A_{it}^{b}\times T_{it}^{c}\times e_{it}$$

where *i* denotes the province, *t* represents time, *α* is a constant and *e* is the error term. Taking the natural logarithm of both sides of Equation (16), Equation (17) can be obtained:

(17) $$Ln(C_{it})=Ln(a)+bLn(P_{it})+cLn(A_{it})+d\ln(T_{it})+v_{i}+\varepsilon_{it}$$

where *v* takes control of the province-specific fixed effects, and the new error term $\varepsilon_{it}$ is the natural logarithm of *e*. In Equation (17), two new variables will be added, which are financial development and the square of financial development, because the current coefficients cannot reflect the relationship between financial development and carbon emissions. The introduction of the financial development aims to determine whether the influence is positive or negative, and the function of the quadratic term is to test whether the inverted-U shape exists. In this model, *P* denotes the variable of the population (POP), *A* is represented by GDP per capita (GDPPC), and *T* stands for the industrial structure (IS). According to [29,91], we remove population from the right-hand side and divide the dependent variable by the population. The detailed equation is shown in Equation (18):

(18) $$Ln(C_{it})=Ln(a)+b^{\prime }Ln(GDPPC_{it})+c^{\prime }Ln(IS_{it})+d^{\prime }Ln(F_{it})+e^{\prime }Ln{\left(F_{it}\right)}^{2}+v_{i}+\varepsilon_{it}$$

In Equation (18) $C_{it}$ denotes the carbon emissions per capita (CEPC), $F_{it}$ measures financial development, and $F_{it}{}^{2}$ denotes the quadratic term of financial development. In addition, in this paper, we also investigate the relationship between the carbon emissions and the seven detailed aspects of financial development: financial size (FSZ), financial structure (FST), financial openness (FOP), financial depth (FDP), financial growth (FGR), financial efficiency (FEF) and financial ecology (FEC). Thus, Equation (18) can be expanded into Equation (19) when measuring such relations:

(19) $$\begin{array}{l} Ln(C_{it})=Ln(a)+b^{\prime \prime }Ln(GDPPC_{it})+c^{\prime \prime }\ln(IS_{it})\\ +d^{\prime \prime }\ln(FSZ_{it})+v_{i}+\varepsilon_{it}\\ \ldots \\ Ln(C_{it})=Ln(a)+b^{\prime \prime }Ln(GDPPC_{it})+c^{\prime \prime }\ln(IS_{it})\\ +j^{\prime \prime }\ln(FEC_{it})+v_{i}+\varepsilon_{it} \end{array}$$

### 3.5. The Pooled Mean Group Estimator

If the panel dataset is stationary, the standard panel regressions on Equation (18) can be applied to estimate effective and consistent parameters. Otherwise, a dynamic model obtained by using an auto regressive distributed lag model (ARDL) needs to be constructed for the panel data with cointegration. The developed ARDL model is a type of auto regressive model of order *p* in the dependent variable *C*, and the form is ARDL (*p*; *q*, *q*, *q*, *q*), as shown in Equation (20):

(20) $$\begin{array}{l} Ln(C_{it})=Ln(a)+\sum_{j=1}^{p}\lambda_{ij}Ln(C_{i,t-j})+\sum_{j=1}^{q}b_{ij}^{\prime }Ln(GDPPC_{i,t-1})\\ +\sum_{j=1}^{q}c_{ij}^{\prime }Ln(IS_{i,t-1})+\sum_{j=1}^{q}d_{ij}^{\prime }Ln(F_{i,t-1})+\sum_{j=1}^{q}e_{ij}^{\prime }Ln{\left(F_{i,t-1}\right)}^{2}+v_{i}+\varepsilon_{it} \end{array}$$

Equation (21) is transformed into a panel error-correction model to enhance the efficiency and is a PMG form. The PMG approach dictates that in estimating the long-run effects and the speed of adjustments to the long run, we must allow the short-run dynamics to be determined for each country. The PMG estimators are consistent and efficient even in the presence of endogenous and non-stationary regressors, which is described in Equation (21):

(21) $$\begin{array}{l} \Delta Ln(C_{it})=\phi_{i}[Ln(C_{i,t-1})-b_{i}^{\phi }Ln(GDPPC_{i,t})-c_{i}^{\phi }Ln(IS_{i,t})-d_{i}^{\phi }Ln(F_{i,t})\\ -e_{i}^{\phi }Ln{\left(F_{i,t}\right)}^{2}]+\sum_{j=1}^{p-1}\lambda_{ij}^{*}\Delta Ln(C_{i,t-j})+\sum_{j=0}^{q-1}b_{ij}^{*}\Delta Ln(GDPPC_{i,t-j})\\ +\sum_{j=0}^{q-1}c_{ij}^{*}\Delta Ln(IS_{i,t-j})+\sum_{j=0}^{q-1}d_{ij}^{*}\Delta Ln(F_{i,t-j})+\sum_{j=0}^{q-1}e_{ij}^{*}\Delta Ln{\left(F_{i,t-j}\right)}^{2}+v_{i}+\varepsilon_{it} \end{array}$$

where $\phi_{i}=\sum_{j=1}^{p}\lambda_{ij}-1$, $\lambda_{ij}^{*}=-\sum_{m=j+1}^{p}\lambda_{im}$, $a_{i}^{\phi }=-\sum_{j=0}^{q}a_{ij}^{\prime }/\phi_{i}$, $b_{i}^{\phi }=-\sum_{j=0}^{q}b_{ij}^{\prime }/\phi_{i}$, $c_{i}^{\phi }=-\sum_{j=0}^{q}c_{ij}^{\prime }/\phi_{i}$, $d_{i}^{\phi }=-\sum_{j=0}^{q}d_{ij}^{\prime }/\phi_{i}$, $e_{i}^{\phi }=-\sum_{j=0}^{q}e_{ij}^{\prime }/\phi_{i}$, $a_{i}^{*}=-\sum_{m=j+1}^{p}a_{im}^{\prime }$, $b_{i}^{*}=-\sum_{m=j+1}^{p}b_{im}^{\prime }$, $c_{i}^{*}=-\sum_{m=j+1}^{p}c_{im}^{\prime }$, $d_{i}^{*}=-\sum_{m=j+1}^{p}d_{im}^{\prime }$, $e_{i}^{*}=-\sum_{m=j+1}^{p}e_{im}^{\prime }$.

In Equation (21), $\phi_{i}$ is the error-correcting speed of adjustment, and with a significantly negative value, the impacts of the independent variables are cointegrated. That is, the long-run mean reversion exists. The long- run influences are represented by $a^{\phi }$, $b^{\phi }$, $c^{\phi }$, $d^{\phi }$ and $e^{\phi }$, respectively. Accordingly, the short-run impact is captured by $a^{*}$, $b^{*}$, $c^{*}$, $d^{*}$ and $e^{*}$.

Based on Binder and Offermanns [92], Equation (21) can be respecified to correct for the cross-sectionally correlated errors as follows:

(22) $$\begin{array}{l} \Delta Ln(C_{it})=\phi_{i}[Ln(C_{i,t-1})-b_{i}^{\phi }Ln(GDPPC_{i,t})-c_{i}^{\phi }Ln(IS_{i,t})-d_{i}^{\phi }Ln(F_{i,t})-e_{i}^{\phi }Ln{\left(F_{i,t}\right)}^{2}]\\ +\sum_{j=1}^{p-1}\lambda_{ij}^{*}\Delta Ln(C_{i,t-j})+\sum_{j=1}^{q-1}b_{ij}^{*}\Delta Ln(GDPPC_{i,t-j})+\sum_{j=1}^{q-1}c_{ij}^{*}\Delta Ln(IS_{i,t-j})+\sum_{j=1}^{q-1}d_{ij}^{*}\Delta Ln(F_{i,t-j})\\ +\sum_{j=1}^{q-1}e_{ij}^{*}\Delta Ln{\left(F_{i,t-j}\right)}^{2}+\sum_{j=1}^{p-1}\bar{\lambda_{ij}^{\gamma }\Delta Ln(C_{i,t-j})}+\sum_{j=1}^{q-1}\bar{b_{ij}^{*}\Delta Ln(GDPPC_{i,t-j})}+\sum_{j=1}^{q-1}\bar{c_{ij}^{*}\Delta Ln(IS_{i,t-j})}\\ +\sum_{j=1}^{q-1}\bar{d_{ij}^{*}\Delta Ln(F_{i,t-j})}+\sum_{j=1}^{q-1}\bar{e_{ij}^{*}\Delta Ln{\left(F_{i,t-j}\right)}^{2}}+v_{i}+\varepsilon_{it} \end{array}$$

where $\sum_{j=1}^{p-1}\bar{\lambda_{ij}^{\gamma }\Delta Ln(C_{i,t-j})}$, $\sum_{j=1}^{p-1}\bar{b_{ij}^{\gamma }\Delta Ln(UR_{i,t-j})}$, $\sum_{j=1}^{p-1}\bar{c_{ij}^{\gamma }\Delta Ln(IS_{i,t-j})}$, $\sum_{j=1}^{p-1}\bar{d_{ij}^{\gamma }\Delta Ln(F_{i,t-j})}$ and $\sum_{j=1}^{p-1}\bar{e_{ij}^{\gamma }\Delta Ln{\left(F_{i,t-j}\right)}^{2}}$ are the cross-sectional average.

## 4. Data and Variables

The research aimed to study the relationship between financial development (FD) and carbon emissions level (CEL) based on a regional analysis by applying the panel data, so we collected data covering 30 provinces in China from 2000 to 2013, considering the data availability. A detailed calculation of the variables is introduced in this section. 

### 4.1. Calculation of Carbon Emissions in Each Province

Currently, fossil fuel combustion and the production of cement are two main sources for carbon emissions in China, so when calculating carbon emissions, this paper considers these two aspects. The most commonly used fossil fuels in China are coal, oil and natural gas, which results in a proportion of 66%, 17.1% and 5.7%, respectively, in total energy consumption; thus, this paper chooses the terminal consumption values of 13 types of main fossil fuels to calculate the cumulative carbon emissions to enhance the accuracy of data, including raw coal, cleaned coal, other washed coal, briquettes, coke, crude oil, gasoline, kerosene, diesel oil, fuel oil, liquefied petroleum gas, refinery gas and natural gas. The detailed calculation equation is described as follows:

(23) $$C_{itf}=\sum_{j=1}^{13}C_{itj}=\sum_{j=1}^{13}M_{itj}\times K_{j}\times q_{j}$$

where $M_{itj}$ represents the physical quantity of the *j*th fossil fuel in the *i*th province in the *t*th year. $K_{j}$ denotes the standard coal efficiency, which can be found in the China Statistical Yearbook (2014) [93], and $q_{j}$ is the carbon emissions coefficient published by IPCC in 2006 [94]. Assuming that the utilization efficiency of fossil fuels is unchanged in the short term, the carbon emissions coefficient of each fossil fuel is constant. 

The level of carbon emissions during the cement production process is also taken into consideration. The calculation is shown in Equation (24):

(24) $$C_{itc}=N_{it}\times a$$

where $N_{it}$ represents the physical quantity of cement in the *i*th province in the *t*th year, and *a* is the carbon emissions factor, which is 0.5272 [95]. The data of the cement output in each province of China are collected from the CEinet Statistics Database [96]. 

### 4.2. Financial Development Index System (FDIS)

Financial development covers a wide range, and it is a challenging and difficult task to measure it accurately. We need to build a comprehensive and scientific index system to describe the regional financial development of China. 

As discussed above, financial development has been measured by using different types of indexes without a unified standard, which will cause measuring errors and influence the reliability of the research results. Specifically, this is why people sometimes obtain completely different conclusions when studying the relationship between FD and CEL. Therefore, this paper constructs an innovative financial development index system to overcome the limitations mentioned above. According to the conception of financial development and existing literature, financial development includes the following seven aspects: financial size, financial structure, financial openness, financial depth, financial growth, financial efficiency and financial ecology. The measurement of each indicator is shown in Table 3. The data are obtained from the China Statistical Yearbook (2001–2014), the Statistical Yearbook of each province of China (2001–2014) and the Almanac of China’s Finance and Banking (2001–2014), respectively.

According to the methods of SGPCA and EWM, the FDIS is established. The integrated weight of each indicator in FDIS is listed in Appendix B, Table A2. 

### 4.3. Other Variables

Table 4 shows two control variables, and the data are collected from the China Statistical Yearbook (2001–2014) and China Industrial Economic Statistical Yearbook (2001–2014).

## 5. Empirical Results and Discussion

### 5.1. Current Situations of Carbon Emissions and Financial Development

The clustering method is applied to divide all 30 provinces into four categories as follows: high emission-high development (HE-HD), high emission-low development (HE-LD), low emission-high development (LE-HD) and low emission-low development (LE-LD).

#### 5.1.1. Current Situations of Carbon Emissions in China

**Figure 3:**
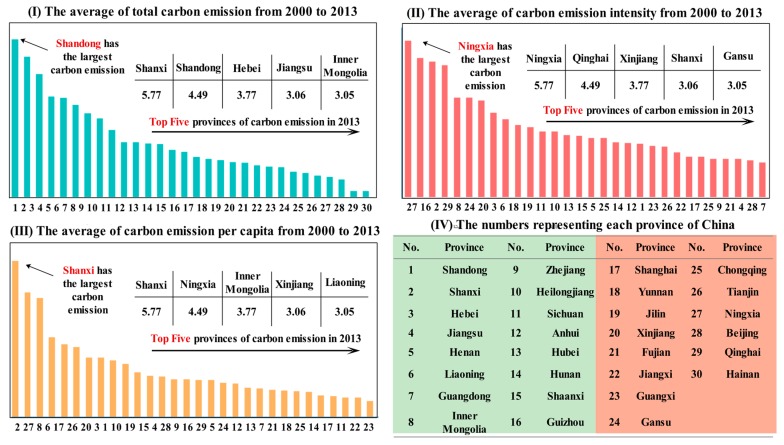
Current situations of carbon emissions level.

From Figure 3I, it can be seen that the carbon emissions of Shandong, Hebei, Shanxi, Henan and Jiangsu dominate the top positions, while the carbon emissions of Qinghai and Hainan are low. Shandong and Shanxi are major provinces that produce coal, and the proportion of coal consumption occupies a large share; thus, the carbon emissions of the two provinces account for nearly 18% of the entire country. 

Figure 3II demonstrates that the carbon emissions intensity (CEI) of Ningxia is nearly three times larger than that of Guangdong. We can conclude that the carbon emissions are related to the economic aggregate, but there is no relationship between CEI and economic aggregate. Instead, CEI is related to the economic development stage and industrial development level. 

Figure 3III shows the changing trend of CEPC. Apart from Shanxi, whose CEPC ranks first in China and is approximately 9.6 times that of Guangxi, the order of the other provinces changes greatly. The result reflects the phenomenon that carbon emissions and energy consumption tend to be centralized in cities where population density is high. Therefore, each large- and middle-scale city has a high potential for reducing carbon dioxide.

#### 5.1.2. Current Situations of Financial Development in China

**Figure 4:**
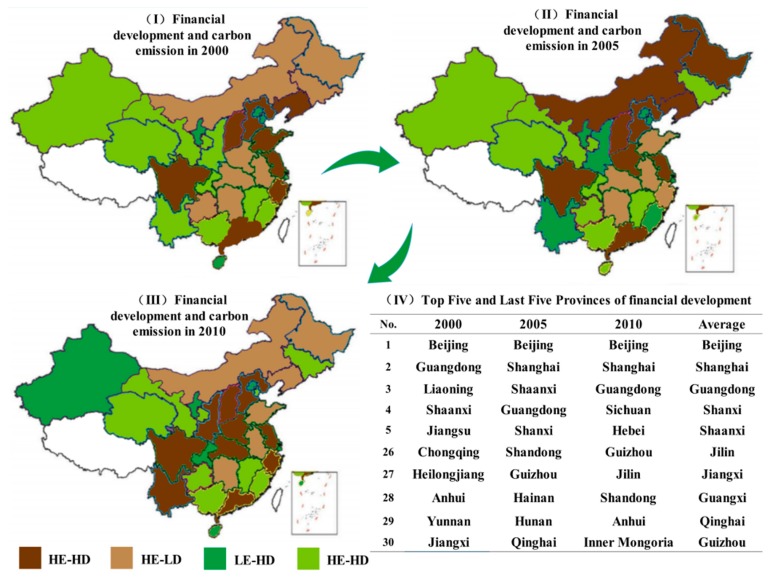
Financial development and cluster results of each province.

Beijing, Shanghai and Guangdong are provinces with the most sophisticated financial development, while Guizhou, Qinghai and Guangxi have the lowest financial development level. According to Figure 4IV, it can be seen that in addition to the top three provinces, the change of financial development in the other provinces is obvious and large. What is worth noticing is that the financial development of Liaoning ranks 3rd; however, in 2010, it ranked 23rd, which reflects a declining tendency. Additionally, the provinces in the north-eastern regions have less developed financial development. In comparison, coastal regions, such as Guangdong, Shanghai and Zhejiang, have strong financial power. Furthermore, the financial development of the western region, including Ningxia and Xinjiang, exhibits a great increasing potential.

#### 5.1.3. Results of the Clustering Analysis

According to the results calculated above, we can rank each province from 1 to 30. Carbon emissions is the *x*-axis, and financial development is the *y*-axis. If the Euclidean distance between a province and the point at the bottom left corner is shorter than the other three corners, this indicates that the province belongs to the HE-HE category. Therefore, the initial clustering points are Beijing (LE-HD), Shanxi (HE-HD), Shandong (HE-LD) and Qinghai (LE-LD). The LE-HD category presents two extremes: financial development in Beijing and Shanghai is advanced; financial development in Xinjiang and Hainan is less advanced. 

With respect to the different years, the changing rule can be calculated as shown in Figure 4. LE-HD can be regarded as the most ideal development mode, and the results show that Shanghai, Xinjiang and Chongqing shift from HE-HD to LE-HD due to the improvement of the production and utilization efficiency. The HE-LD mode is the least ideal. Both Liaoning and Shandong transfer from HE-HD to HE-LD, reflecting problems during the process of financial development. From the perspective of carbon emissions, Shanxi shifts from LE-HD to HE-HD, indicating that the financial development of Shanxi has been at a high level but at the expense of the environment. Although the HE mode can meet the requirements of sustainable development, it cannot be proven that LE is sustainable. Therefore, provinces should adopt effective measures to reduce carbon emissions while promoting economic development. 

### 5.2. Impact of Financial Development on Carbon Emissions

The results of cross-sectional dependence (CD), panel unit root test and panel cointegration tests are shown in Appendix C. This section will discuss the impact of financial development on the CEL in detail. 

#### 5.2.1. The Overall Impact 

Table 5 shows that the long-term adjustment coefficient of the error correction model is negative, which is in accordance with the principle of reverse correction. Additionally, the CDFE model passes the significance test at the 1% level, which also proves the rationality of the model. In general, the results in Table 5 demonstrate that financial development will promote CEL. A detailed analysis is as follows:

(1) For the long-term impact, the coefficients of financial development on CEPC are 0.291, which is statistically significant. Additionally, the coefficient of FD^2^ is negative, which is −0.321, proving the inverted U-shape relationship between financial development and CEPC in China. The results indicate that, at the present stage, the development of finance is at the expense of the environment.
(2) Compared with long-term influence, the short-term coefficient of FD on the CEPC is 0.375. The results show that the short-term influence degree is larger than the long-term impact; therefore, the inhibiting effect of the FD on CEL requires a long time to be realized. This conclusion is valuable because it suggests that policy makers consider the long-term influence when developing policies.
(3) Finally, for the overall impact of other variables, the GDPPC can facilitate CEPC. The industrial structure contributes to CEPC with a coefficient of 0.310. The results fully reflect the characteristics of high output value and low production efficiency during the industrialization process.

#### 5.2.2. The Impact on Different Regions

China is a vast territory with different financial development levels, levels of GDP per capita and industrial structures among the eastern, middle, western and north-eastern regions. The difference in each area is complicated and distinctive. To explore the regional difference, China is divided into four regions as shown in Appendix D, Table A6 [64]. The number that represents each province is shown in Figure 3IV. The results of Table 6 are analysed as follows:

(1) Eastern region. FD constrains CEL in the long term. That is, if the financial development increases 1%, CEPC decreases 0.374%. Meanwhile, the coefficient of FD^2^ is both negative and significant, indicating an inverted U-shape relationship between financial development and CEL [97]. Financial development in the eastern region tends to be mature, so it can constrain CEL. GDPPC can cause carbon emissions to increase, leading to a rise of energy consumption and demand. The results of other variables fully prove that the industrial process of the eastern region contains technological advancement and improvements in production efficiency, which are caused by the factor reallocation [19].
(2) Middle region. The impact is statistically significant at the 1% level with positive coefficients of 0.391. The GDPPC increases CEPC with a coefficient of 0.326. The industrial structure has a significant and positive impact on CEL. In the short term, the coefficient is larger, indicating that the middle region needs time to achieve the carbon emissions reduction.
(3) Western region. The long-term coefficient of FD on CEL in western region is negative and significant, but the absolute value, 0.461, is larger than the middle region. Thus, with the improvement of the financial development level, carbon emissions will increase at a greater magnitude than the middle region. The GDPPC can affect the carbon emissions level positively at the 1% level. The western region is in a start-up stage, and the region needs to improve the investment environment, increase openings, attract social capital and participate actively in western development and establishment.
(4) North-eastern region. The influence is positive and significant and exceeds the middle region at a value of 0.698. The influence of other variables is similar to that in the western region. In the short term, the impact of FD on CEPC is 0.429. As a traditional industrial base, the north-eastern region has an extensive economic development pattern. The accumulated deep contradiction is gradually becoming obvious, causing low utilization efficiency in each resource and condition. Financial development proceeds at the cost of environment.

#### 5.2.3. The Impact on Different Stages

To increase more constructive suggestions on achieving the goal of carbon emissions reduction in China in 2020, this section studies the relationship between FD and CEPC in the 10th FYP, 11th FYP and 12th FYP. A detailed analysis of Table 7 is listed below:

(1) During the 10th FYP (2001–2005), stimulated by industrialization, urbanization and internationalization, the Chinese economy achieved rapid expansion, and the industrial structure tended toward high input, energy consumption and pollution. At that time, financial development promoted CEL, and each 1% increase of financial development caused a 0.816% increase in CEPC. Therefore, financial development aggravates the carbon emissions in this stage because of the outdated production capacity, increase of enterprises with high energy consumption, lagging production technology and low production efficiency [18].
(2) The 11th FYP was a transformative period for Chinese carbon emissions with remarkable progression of low-carbon development. The increase of carbon emissions was effectively alleviated, and the increasing tendency of CEL begins to decrease. Therefore, during this time, the economic development mode changes from an investment-led pattern to a science-led pattern. Financial development can promote CEL with a coefficient of 0.569. This confirms that although financial development can still increase carbon emissions, the impact effect greatly decreases. On the other hand, it shows that the strategies of energy conservation and emission reduction are effective and can contribute to low-carbon development.
(3) The 12th FYP is a key stage for China to develop its low-carbon economy. China further adjusted its energy structure, established an energy system, changed the industrial structure, promoted the development of low-carbon industries, and developed low-carbon technology. During this time, FD promoted CEL with a lower impact magnitude, which indicates that the low-carbon development mode accelerated the independent innovation process. The expansion of financing channels, continuous perfection of the carbon trade mechanism and improvement of technological research abilities are beneficial for China in realizing a low-carbon economy.

#### 5.2.4. The Impact of Different Aspects of Financial Development on CEL

The empirical results in Table 8 show that the influence of seven specific aspects of financial development are not the same. Due to the limitation of the length of this paper, we only list the long-run results estimated by CDFE. From a national perspective, FSZ, FOP and FDP will increase carbon emissions, while FEF and FEC will help constrain the CEL. In each region, the influences are also different. Therefore, it is necessary to analyse each aspect and adjust the policies accordingly. The results are summarized in Table 9, and the influences of FST and FGR [98] are not statistically significant, so we will not discuss the two indicators.

## 6. Conclusions

**Figure 5:**
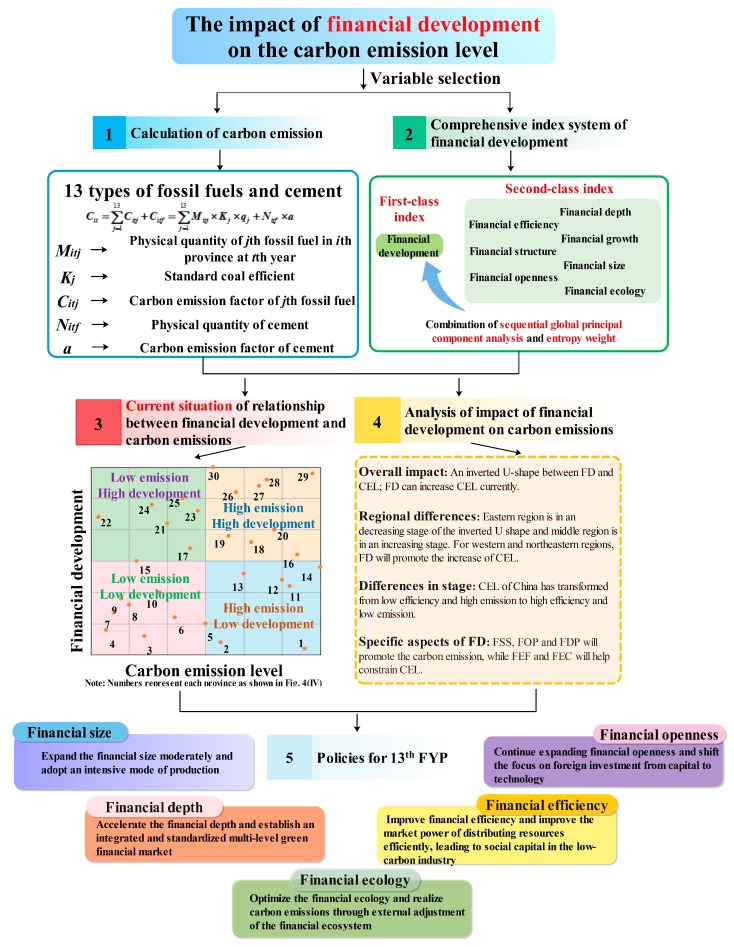
Summary of the research results.

This paper aimed to study their relationship more deeply and thoroughly to theoretically and empirically contribute to carbon emissions reduction in China. The detailed conclusions are shown in Figure 5. Initially, a method for analysing carbon emissions considered 13 types of fossil fuels and cement, and a comprehensive index system to measure financial development is built based on the combination of a sequence global principal component analysis and the entropy weight method. Then, the extended STIARPAT model and dynamic error-correction model for panel data are employed to research the impact from the perspective of the entire country, regional differences and stage differences. Additionally, we also divide financial development into seven specific aspects to study their influences on carbon emissions.

The main research conclusions are summarized as follows: (1) From a national perspective, whether measuring CEL by using CEPC, there is an inverted U-shape between FD and CEL. Currently, the development of finance can increase carbon emissions greatly, which indicates that financial development plays a larger role in stimulating consumption and expanding the investment scale than in the technological advancement and structure updating; (2) The eastern region is in a decreasing stage in an inverted-U shape, and the middle region is in an increasing stage. For both western and north-eastern regions, the current financial development will promote the increase of carbon emissions; (3) During the 10th FYP, the increase of energy consumption leads to improvement of carbon emissions. The detrimental impact of financial development on CEL tends to be weaker during the 11th FYP because the economic growth mode begins to transform from an extensive to intensity mode. For the 12th FYP, with the establishment of a resource-saving and environmentally friendly economic system, financial development will gradually constrain carbon emissions. In summary, carbon emissions in China have transformed from low efficiency and high emissions to high efficiency and low emissions; (4) Financial size, financial openness, and financial depth can facilitate the increase of carbon emissions; however, in comparison, financial efficiency and financial ecology can effectively constrain the carbon emissions level. The influences of financial structure and financial growth are not significant. 

To sum up, we have proposed a novel approach and conducted a thorough and comprehensive study to analyse the impact of financial development on the carbon emissions level, which will be helpful for policy makers to develop corresponding policies to realize the goal of carbon emissions reduction and achieve sustainable development in China.

Based on the conclusions above, policies can be proposed for the 13th FYP in China to balance the relationship between financial development and the carbon emissions level. It can be seen that carbon emissions will be affected by financial development in terms of financial size, financial openness, financial depth, financial efficiency and financial ecology. Therefore, the Chinese government needs to propose reasonable and detailed suggestions and policies according to the abovementioned factors of financial development and focus on the gaps between developed regions and less developed regions by strengthening technological cooperation and adjusting the measures to local conditions. The detailed policies on controlling carbon emissions are listed below:

◆ Expand the *financial size* moderately and adopt an intensive mode of production

Increasing financial size will lead to the increase of carbon emissions; however, this does not mean that the expansion of financial size prevents carbon emissions reduction. In contrast, expanding the financial size reasonably can increase the fund supply, which can help reduce the costs of introducing low-carbon technologies and increasing the motivation of enterprises to reduce carbon emissions. Therefore, the idea is to adjust the production mode of enterprises from an extensive pattern to an intensive pattern and eliminate companies with high emissions and overcapacity. The government should intensify supervision and implement regulations and rules promoting low-carbon development to avoid uncontrolled expansion of the financial size. 

◆ Continue expanding *financial openness* and shift the focus on foreign investment from capital to technology

Expanding financial openness is beneficial, but the low entrance threshold and weak national competitiveness will result in low FDI investment quality, which can further increase unnecessary carbon emissions. Therefore, while expanding financial openness, the barriers to entry for the market should be improved. Additionally, developing countries also need to increase the appeal to FDI by reinforcing competitiveness, and the focus of foreign investment should be on the technology level instead of the capital scale. Finally, the government should guide foreign investment in the low-carbon industry and encourage financial creation, such as carbon finance, carbon bonds and so on. 

◆ Accelerate the *financial depth* and establish an integrated and standardized multi-level green financial market

First, extending bank reform and the financial industry is beneficial to attracting investment in environmental protection facilities and technology, are necessary. Second, financial institutions should increase green-credit loans and encourage more investment in the environmental protection industry. Low-carbon enterprises can realize indirect financial gains through green credit and intermediary services of carbon finance. The government also needs to increase its support of the low-carbon industry and expand the direct financing of low-carbon enterprises by building green channels for their listing and establishing a carbon exchange market to guide capital to the low-carbon industry. 

◆ Improve *financial efficiency* and improve the market power of distributing resources efficiently, leading to social capital in the low-carbon industry

On the one hand, both the conversion rate of deposit and the loan and utilization efficiency of financial capital should be improved. Financial institutions should track the management of enterprise capital, and more capital should be used to change the production technology of enterprises. On the other hand, the low-carbon consumption of residents should be improved so they purchase more low-energy consuming to optimize the financial environment of consumption. 

◆ Optimize the *financial ecology* and realize carbon emissions through external adjustment of the financial ecosystem

First, the government should actively improve the financial ecological environment instead of passively meeting the requirements of economic activity. Accelerating improvement of financial ecology is beneficial for promoting energy conservation and emissions reduction of the real economy and achieving a low-carbon pattern earlier. Second, a certification system for low-carbon products should be created to increase awareness of low-carbon consumption by residents. Financial institutions can provide detailed channels to reduce carbon emissions by developing new carbon-related products. Finally, the government should encourage financial institutions to perform financial innovation of low-carbon products to offer companies and individual customers more opportunities to invest in the low-carbon industry, supporting carbon emissions reduction and low-carbon economic development.

## Figures and Tables

**Table 1:** Summary of STIRPAT model and factor decomposition analysis.

Country	Year	Influencing Factors	Estimated Methods	Equation	Ref.
**Factor Decomposition Model**					
EU-28	2000–2012	Level of activity, electricity intensity, electricity trade, efficiency of electricity generation and fuel mix	LMDI-I method with the use of logarithmic mean weight functions	$C_{t}=A_{t}\frac{EC_{t}}{A_{t}}\frac{EP_{t}}{EC_{t}}\sum_{i}\frac{F_{i,t}}{EP_{i,t}}\frac{F_{i,t}}{F_{t}}f_{i,t}=A_{t}I_{t}T_{t}\sum_{i}e_{i,t}s_{i,t}f_{i,t}$	[14]
United States	2005–2025	CO_2_ intensity of energy use, energy intensity of output, structural change, GPD per capita, population	Kaya identity additive Logarithmic Mean Divisia Index (LMDI) method	$G_{k}=\frac{G_{k}}{E_{k}}\cdot \frac{E_{k}}{Q_{k}}\cdot \frac{Q_{k}}{Q}\cdot \frac{Q}{P}\cdot P,\;G=\sum_{i}\frac{G_{i}}{FFE}\cdot \frac{FFE}{E}\cdot \frac{E}{Q}\cdot \frac{Q}{P}\cdot p$	[15]
China	2000–2012	Energy structure, intensity, energy efficiency, economic development, population	Logarithmic Mean Divisia Index (LMDI) method	$C^{t}=\sum_{i,j}C_{ij}^{t}=\sum_{i,j}\frac{E_{ij}^{t}}{E_{j}^{t}}\cdot \frac{C_{ij}^{t}}{E_{ij}^{t}}\cdot \frac{E_{j}^{t}}{Y_{j}^{t}}\cdot \frac{Y_{j}^{t}}{P_{j}^{t}}\cdot P_{j}^{t}=\sum S_{ij}^{t}I_{ij}^{t}F_{j}^{t}R_{j}^{t}P_{j}^{t}$	[16]
China	2001–2011	Energy mix change, potential energy intensity change, economic activity, energy usage efficiency, energy saving technology change, GDP technical efficiency, GDP technology change	the multiplicative LMDI method	$C^{s}=\sum_{j}C_{j}^{s}=\sum_{j}\frac{C_{j}^{s}}{E_{j}^{s}}\times \frac{E_{j}^{s}}{E^{s}}\times \frac{E^{s}}{Y^{s}}\times Y^{s},s\in \{0,t\}$	[17]
**STIRPAT model**					
China	1990–2012	Percentages of population employed in secondary and tertiary industries, percentage of the population living in urban areas, shares of the added value of secondary and tertiary industry to the GDP, rural-urban income gap, the cultivated land area occupied by construction	Fixed effects (FE), the feasible generalized least squares (FGLS) and the linear regression with Driscoll–Kraay standard errors (DK)	$\begin{array}{l} Ln(EC_{it})=a_{0}+a_{1}Ln(EP2_{it})+a_{2}Ln(EP3_{it})+a_{3}Ln(URBA_{it})\\ +a_{4}Ln(URI_{it})+a_{5}Ln(INDU_{it})+a_{6}Ln(TERT_{it})\\ +a_{7}Ln(LAND_{it})+\varepsilon_{lit} \end{array}$	[18]
China	1995–2010	Population, GDP per capita, tertiary industry value, secondary industry output value, FDI, energy consumption	Fixed effects (FE), linear regression with Newey-West standard errors (N-W), panel-corrected standard errors (PCSE), and Driscoll-Kraay standard errors (DK), feasible generalized least squares (FGLS)	$\begin{array}{l} \ln CO_{2it}=a_{0}+a_{1}\ln P_{it}+a_{2}\ln A_{it}+a_{3}\ln T_{it}+a_{4}\ln S_{it}\\ +a_{5}\ln FDI_{it}+a_{6}\ln URB_{it}+e_{it} \end{array}$	[19]
China	1980–2010	Total population, urbanization level, GDP per capita, technology, industrialization, service, foreign trade degree, energy consumption structure	Ridge regression with biased estimates	$\begin{array}{l} \ln I=a_{0}+a_{1}\ln P_{s}+a_{2}\ln P_{c}+a_{3}\ln A+a_{4}\ln T+a_{5}\ln G+a_{6}\\ \times \ln F+a_{7}\ln W+a_{8}\ln O \end{array}$	[20]
China	1990–2008	GDP per capita, industrial structure, population, urbanization rate, technology level, energy consumption	Partial least squares (PLS) regression, linear regression	$\ln C_{t}=a+b\ln A+c\ln IS+d\ln P+m\ln R+n\ln T+e_{t}$	[21]
**CKC/EEO model**					
Morocco	1971–2011	GDP per capita, trade openness, electricity consumption per capita	Vector error correction mechanism (VECM)	$\begin{array}{l} \log CO_{t}=\beta +\beta_{1}\log PC_{t}+\beta_{2}\log PCS_{t}\\ +\beta_{3}\log EC_{t}+\beta_{4}\log TR_{t}+\varepsilon_{t} \end{array}$	[25]
China	2000–2013	GDP per capita, energy intensity, urbanization level	Semi-parametric panel fixed effects regression supplemented with traditional parametric regression estimation method	$\begin{array}{l} \ln CE_{ijt}=\alpha_{i}+\beta_{1}\ln A_{it}+\beta_{2}{\left(\ln A_{it}\right)}^{2}+\beta_{3}\ln EI_{it}\\ +\beta_{4}\ln UR_{it}+T_{t}+\varepsilon_{it} \end{array}$ $\begin{array}{l} \ln CE_{ijt}=\alpha_{i}+\beta_{1}\ln A_{it}+\beta_{3}\ln EI_{it}+\beta_{4}\ln UR_{it}\\ +\beta_{5}{\left(\ln UR_{it}\right)}^{2}+T_{t}+\varepsilon_{it} \end{array}$	[26]
Croatia	1992–2011	GDP	Autoregressive distributed lag model (ARDL), dynamic ordinary least squares (DOLS), fully modified ordinary least squares (FMOLS)	$CO_{2t}=\beta_{0}+\beta_{1}{\left(GDP\right)}_{t}+\beta_{2}{\left(GDPSQ\right)}_{t}+\varepsilon_{t}$	[27]
164 countries	1960–2011	GDP per capita	Ordinary least squares (OLS)	$lnE_{it}=\alpha_{i}+\beta_{1t}\ln Y_{it}+\beta_{2t}{\left(\ln Y_{it}\right)}^{2}+\varepsilon_{it}$	[28]
Korea	1978–2007	Income, energy consumption, electricity production (thermal power, nuclear)	Autoregressive distributed lag model (ARDL), ordinary least squares (OLS)	$\begin{array}{l} \ln{\left(CO_{2}\right)}_{t}=a_{0}+a_{1}\ln Y_{t}+a_{2}{\left(\ln Y_{t}\right)}^{2}+a_{3}\ln EN_{t}\\ +a_{4}{THR}_{t}+\varepsilon_{t} \end{array}$ $\begin{array}{l} \ln{\left(CO_{2}\right)}_{t}=b_{0}+b_{1}\ln Y_{t}+b_{2}{\left(\ln Y_{t}\right)}^{2}+b_{3}\ln EN_{t}\\ +b_{4}NUR_{t}+\mu_{t} \end{array}$	[32]
Southeast Asian Nations	1997–2009	Real income per capita, energy use per capita	Autoregressive distributed lag model (ARDL), ordinary least squares (OLS)	$LE_{t}=\beta_{0}+\beta_{1}LY_{t}+\beta_{2}{\left(LY_{t}\right)}^{2}+\beta_{3}LEN_{t}+\varepsilon_{t}$	[33]
South Africa	1971–2010	Energy use, GDP per capita	Autoregressive distributed lag model (ARDL), ordinary least squares (OLS)	$y_{t}=\beta^{+}x_{t}^{+}+\beta^{-}x_{t}^{-}+u_{t}$	[34]

**Table 2:** Summary of studies of the relationship between financial development and carbon emissions.

Time	Countries	Carbon Emissions	Financial Development	Method	Relationship	Ref.
1975–2011	Indonesia	CO_2_ emission per capita	Real domestic credit to private sector per capita	Unit root/ARDL	Financial development condenses carbon emissions and inverted-U shaped relationship is confirmed between financial development and carbon emissions.	[45]
1975–2011	UAE	CO_2_ emission per capita	Domestic credit to private sector	Unit root/Co-integration	Find an inverted U-shaped relationship between financial development and CO_2_ emissions.	[2]
1971–2011	Malaysia	CO_2_ emission per capita	Real domestic credit to private sector per capita	ARDL/VECM	Financial development can play positive and significant role in combating environmental degradation in the country.	[69]
1953–2006	China	CO_2_ emission per capita	Ratio of deposit liabilities to nominal GDP, ratio of credit to private sector to nominal GDP, ratio of commercial bank assets to the sum of commercial bank and central bank assets, foreign assets plus the foreign liabilities as a share of GDP	ARDL	Financial development has led to a decrease in environmental pollution.	[70]
1980–2012	Gulf Cooperation Council Countries	CO_2_ emission per capita	Domestic credit available to the private sector as share of GDP	PVECM	Financial development was found to reduce CO_2_ emissions in the long-run; Financial development would continue to impact CO_2_ emissions little magnitude into the future.	[71]
1980–2008	19 countries	CO_2_ emission per capita	Broad money, domestic credit provided by banking sector, and the domestic credit to private sector	Granger causality test	CO_2_ emission affected the financial development based on the long run causal relationship and the positive short run causal relationship.	[72]
1980–2008	Sub Saharan African countries	Carbon emissions	Broad money, the domestic credit to private sector	Granger causality test	CO_2_ emission had a long run impact and a positive causal relationship on the financial development. The financial development indicators had a positive causal relationship with the CO_2_ emission.	[73]
1985–2011	40 countries	Carbon emissions	Domestic credit to private sector	EKC/PVECM	Increases in financial development decrease carbon emissions.	[74]
1960–2007	Turkey	CO_2_ emission per capita	Domestic credit to private sector	ARDL/Granger causality test/VEC	There is a long-run relationship between per capita carbon emissions and financial development. Financial development variable has no significant effect on per capita carbon emissions in the long run.	[75]
1971–2011	Pakistan	CO_2_ emission per capita	Financial intermediation development: Total Credit, Private Sector Credit; Stock market development: Stock market capitalization, Stocks traded/turnover; Foreign direct Investment	ARDL//VECM	CO_2_ emission per capita is co-integrated with financial development; Financial development contributes to the increase of CO_2_ emission; FDI had a unidirectional causal relationship with emissions.	[76]
1960–2010	Turkey	CO_2_ emission per capita	Real domestic credit to private sector per capita	Granger causality test	There is a unidirectional relationship between financial development and carbon emissions.	[77]
1971–2008	India	Carbon emission	Domestic credit to private sector	ARDL/Granger causality test	Financial development has a long-run positive impact on carbon emissions. There is a long-run unidirectional causality running from financial development to carbon emissions and energy use.	[78]
1985–2005	World	CO_2_ emission per capita	Financial openness	EKC/Quantile regression model	More financial openness does not appear to reduce the carbon emissions.	[79]
1965–2008	South Africa	CO_2_ emission per capita	Real domestic credit to private sector per capita	Unit root/ARDL/ECM	Banking sector development that is per capita access to domestic credit of private sector help to achieve lower CO_2_ per capita emissions.	[1]
1994–2009	China	Carbon emissions	Financial intermediation scale, financial intermediation efficiency, stock market scale, stock market efficiency, foreign direct investment	VAR/Granger causality test/VECM	The influence of financial intermediation scale is the largest with weaker efficiency’s influence. Stock market scale has relatively larger influence on carbon emissions with limited efficiency. FDI exerts the least influence on the change of carbon emissions.	[56]
1976–2009	Vietnam	CO_2_ emission per capita	Real financial direct investment per capita	Co-integration/Granger causality test	The FDI is found to be negatively affecting CO_2_ emissions.	[80]
1989–2011	13 European/12 Asia and Oceania	Carbon emissions	The ratio of domestic credit to the private sector to GDP, and the stock traded turnover ratio	PVAR	CO_2_ shocks on both credit and stock markets are insignificant.	[11]

**Table 3:** Summary of seven specific aspects of the financial development.

Criteria Level	Factor Level	Measuring Level
FSZ	Financial asset	X_1_: Gross of banking assets, Security assets and Premium income
	Financial institutions	X_2_: Number of insurance and Security institutions
	Financial professionals	X_3_: Total number of financial professionals
FST	Financial industrial structure	X_4_: Banking assets/Financial assets
	Internal structure of banking industry	X_5_: Deposit/Loan
FOP	Capital flow liberalization	X_6_: FDI/Total investment in fixed assets
	Localization of foreign financial service	X_7_: Gross of FDI in financial industry
FDP	Finacialization	X_8_: Gross of deposit and loan of financial institutes/GDP
	Financial depth rate	X_9_: Financial assets/GDP
	Foreign direct investment depth rate	X_10_: FDI/GDP
FGR	Financial increasing	X_11_: Increasing speed of RMB deposit of financial institutions
		X_12_: Increasing rate of RMB deposit of residents
	Capital formation speed	X_13_: Gross capital formation/GDP
	Insurance density	X_14_: Premium per capita
	Insurance depth	X_15_: Premium income/GDP
FEF	Macroscopic allocation efficiency	X_16_: Transform rate of saving to investment
		X_17_: Capital formation rate
	Saving rate	X_18_:Saving/Disposal personal income
	Marginal productivity of capital	X_19_: GDP growth/Total fixed asset investment
FEC	Institutional environment	X_20_: Variety of distribution
	Intermediary services	X_21_: Rate of patent authorization and pending
	Social credit system	X_22_: Individual credit

**Table 4:** List of other variables.

Name	Variable Measure	Symbol	Expected Sign	Economic Implications
GDP per capita	GDP/total population	GDPPC	+/−	The influence is uncertain. According to EKC, carbon emissions would increase first with the rise of GDP per capita and then show a declining trend after a certain threshold value is reached.
Industrial structure	Total industrial output value/GDP	IS	+/−	The process of industrialization includes technological advancement, which can redistribute the production factors and improve the production efficiency. However, in the early stages of industrialization, the carbon emissions will increase.

Notes: (1) + indicates that the impact is positive; (2) − indicates that the impact is negative.

**Table 5:** Results of overall impact of FD on CEL.

	Long-Run Coefficients				Short-Run Coefficients			
	CMG	CPMG	CDFE		CMG	CPMG		CDFE
GDPPC	0.228 (0.452)	0.214 *** (3.251)	0.293 *** (7.320)	GDPPC	−0.491 (−0.273)	0.293 (1.193)		0.312 (0.685)
IS	−1.393 ** (−1.982)	0.344 *** (5.619)	0.310 ** (2.020)	IS	0.219 * (1.920)	0.174 ** (2.327)		−0.258 (−0.531)
FD	−0.729 (−0.308)	0.986 * (1.830)	0.291 *** (8.792)	FD	0.154 (0.983)	−0.215 *** (−7.170)		0.375 ** (2.290)
FD^2^	2.175 (0.835)	0.252 *** (3.714)	−0.321 *** (−4.832)	FD^2^	−0.352 (−0.159)	0.719 (1.024)		0.907 (0.925)
Error correction	−0.286 * (−1.992)	−0.491 ** (−2.218)	−0.219 *** (−3.890)	Constant	0.912 (0.014)	2.566 *** (4.376)		4.474 *** (4.140)
Number of obs	390			Hausman test	1		Prob ≥ 1.000	
Number of groups	30				2		Prob ≥ 0.989	
Log Likelihood	881.343				3		Prob ≥ 1.000	

Notes: (1) ***, **, and * denote a significance of 1%, 5%, and 10%, respectively; (2) Hausman test 1, H0: The CPMG estimator is preferred than CMG estimator; (3) Hausman test 2, H0: The CDFE estimator is preferred than CPMG estimator; (4) Hausman test 3, H0: The CDFE estimator is preferred than CMG estimator.

**Table 6:** Results of impact in difference regions.

		Eastern Region				Middle Region			Western Region			Northeastern Region		
		CMG	CPMG		CDFE	CMG	CPMG	CDFE	CMG	CPMG	CDFE	CMG	CPMG	CDFE
Long-run coefficients	GDPPC	0.347 * (−1.832)	0.314 ** (2.130)		0.224 *** (3.261)	0.329 * (1.922)	−0.131 (−0.840)	0.326 ** (2.281)	0.318 ** (2.175)	0.205 *** (4.435)	0.342 *** (7.123)	−0.279 *** (−4.328)	−0.273 ** (−2.251)	−0.125 *** (6.764)
	IS	−0.358 ** (−6.486)	−0.208 * (−1.945)		−0.352 ** (−2.471)	0.246 *** (2.929)	−0.253 * (−1.842)	0.510 (0.529)	0.353 ** (2.334)	0.411 (1.150)	0.261 *** (4.205)	0.622 ** (2.335)	0.832 * (1.8192)	0.741 *** (3.935)
	FD	−0.335 * (−1.920)	0.427 (1.207)		−0.374 *** (−3.238)	0.521 *** (4.837)	−0.581 (−1.428)	0.391 *** (4.519)	0.613 ** (2.029)	0.426 * (1.992)	0.461 *** (4.298)	0.633 * (1.826)	0.741 *** (3.514)	0.698 ** (2.279)
	FD^2^	−0.348 ** (−2.205)	−0.359 ** (−2.275)		−0.258 *** (−5.324)	−0.762 *** (−4.732)	0.164 * (1.938)	−0.873 ** (−2.134)	−0.523 ** (−2.490)	−0.741 (−0.148)	−0.915 *** (−3.904)	−0.674 ** (−2.435)	−0.733 (−1.532)	−0.582 * (-1.973)
Error correction		0.138 * (1.831)	−0.427 *** (−2.794)		−0.416 *** (−4.190)	−0.301 ** (−2.13)	−0.315 ** (−2.143)	−0.251 *** (−6.237)	−0.379 (−1.146)	−0.291 ** (−2.101)	−0.313 *** (−3.850)	−0.304 ** (−2.345)	−0.135 *** (−1.453)	−0.421 *** (−2.890)
Short-run coefficients	GDPPC	0.136 * (1.841)	−0.142 (−1.219)		−0.115 * (−1.893)	−1.750 (−1.28)	−0.019 (−0.928)	−0.225 (−0.451)	0.404 *** (2.845)	−0.897 (−1.320)	0.461 ** (2.354)	0.969 (1.354)	−0.437 * (−1.355)	0.234 * (2.014)
	IS	0.480 * (1.971)	0.179 ** (1.324)		−0.261 ** (−2.436)	0.220 ** (2.401)	−0.251 * (−1.840)	0.391 (0.202)	−0.451 ** (−2.142)	0.029 (0.640)	−0.361 * (−1.882)	0.372 (1.462)	0.401 ** (2.445)	0.235 ** (2.133)
	FD	−0.312 ** (−2.045)	−0.364 * (−1.813)		−0.334 ** (−2.201)	0.254 *** (−5.605)	0.829 (1.340)	0.913 (0.251)	0.542 *** (5.290)	0.490 (0.270)	0.516 ** (1.346)	0.039 (0.904)	0.910 (0.20)	0.429 * (1.821)
	FD^2^	−0.231 * (1.841)	−0.532 ** (2.289)		−0.237 ** (−2.184)	−0.284 * (−1.784)	−0.841 (−1.129)	−0.418 (−0.582)	−0.031 (−0.340)	−0.158 ** (−2.184)	−0.917 * (−1.942)	−0.114 * (−1.801)	−0.994 (−0.301)	−0.151 * (−1.794)
Constant		−0.081 *** (−4.000)	0.034 ** (2.163)		0.083 *** (3.901)	0.072 *** (4.132)	0.262 ** (2.214)	0.086 ** (2.245)	0.031 ** (2.051)	−0.048 * (−1.910)	0.071 *** (4.406)	0.619 *** (3.410)	0.381 ** (2.400)	0.212 *** (4.137)
Number of obs		130				78			143			39		
Number of groups		10				6			11			3		
Hausman test		1		Prob ≥ 1.000		Prob ≥ 1.000			Prob ≥ 1.000			Prob ≥ 1.000		
		2		Prob ≥ 0.990		Prob ≥ 0.991			Prob ≥ 0.993			Prob ≥ 0.998		
		3		Prob ≥ 1.000		Prob ≥ 1.000			Prob ≥ 1.000			Prob ≥ 1.000		

Notes: (1) ***, **, and * denote a significance of 1%, 5%, and 10%, respectively; (2) Hausman test 1, H0: The CPMG estimator is preferred than CMG estimator; (3) Hausman test 2, H0: The CDFE estimator is preferred than CPMG estimator; (4) Hausman test 3, H0: The CDFE estimator is preferred than CMG estimator.

**Table 7:** Results of FD on CEL in different stages.

		10th Five-Year Plan				11th Five-Year Plan			12th Five-Year Plan		
		CMG	CPMG		CDFE	CMG	CPMG	CDFE	CMG	CPMG	CDFE
Long−run coefficients	GDPPC	0.235 * (1.941)	0.248 ** (2.193)		0.253 *** (4.972)	0.258 ** (2.349)	0.241 *** (4.153)	0.292 *** (4.513)	0.150 ** (2.214)	−0.112 *** (−2.839)	−0.142 *** (−3.514)
	IS	0.624 ** (2.295)	0.427 *** (4.512)		0.720 ** (1.986)	0.449 *** (3.395)	0.316 * (1.912)	0.552 ** (2.215)	0.241 ** (2.127)	0.051 (0.990)	0.301 ** (2.198)
	FD	0.793 *** (4.417)	0.774 ** (2.249)		0.816 *** (2.995)	0.5132 ** (2.310)	0.414 *** (3.154)	0.569 ** (2.114)	0.325 ** (2.117)	0.350 *** (5.214)	0.257 *** (4.146)
	FD^2^	−0.418 ** (−2.251)	−0.162 (−0.829)		−0.298 ** (−2.263)	−0.172 (−1.880)	−0.203 (−0.619)	−0.283 (−0.354)	0.682 (0.292)	−0.541 (−0.430)	0.823 (0.514)
Error correction		−1.933 ** (−1.923)	−0.422 ** (−2.125)		−0.946 *** (−5.959)	−0.354 *** (−3.719)	−0.422 ** (−2.124)	−0.481 *** (−3.081)	−1.293 ** (−1.904)	−0.351 (−0.980)	−1.412 *** (−5.523)
Short−run coefficients	GDPPC	0.301 (0.482)	0.461 (1.201)		−0.439 *** (−4.329)	0.719 (0.343)	0.193 ** (2.273)	0.415 (0.911)	−0.214 * (−1.892)	0.183 ** (2.091)	−0.280 ** (−2.142)
	IS	0.293 * (1.982)	0.923 (0.924)		0.142 * (1.842)	0.993 (0.030)	0.219 *** (2.906)	2.327 (0.092)	−0.011 (−0.203)	−0.383 (−0.330)	−0.282 (−1.231)
	FD	0.192 * (1920)	0.012 (1.021)		0.169 * (1.981)	−0.264 (−0.239)	0.335 (0.349)	−0.243 ** (−2.211)	−1.001 (−0.032)	0.383 (0.590)	−0.342 * (−1.953)
	FD^2^	−0.213 (−0.781)	−0.258 * (−1.811)		−0.213 * (−1.851)	0.232 * (1.914)	−0.914 (−1.228)	0.839 (1.019)	0.928 (1.348)	−0.830 (−0.921)	0939 (1.282)
Constant		0.021 * (1.892)	0.355 (0.091)		−0.475 * (−1.920)	0.439 (1.026)	−0.244 ** (−2.248)	0.214 ** (2.044)	0.312 (0.720)	−1.883 *** (−4.028)	1.251 ** (2.122)
Number of obs		120				120			90		
Number of groups		30				30			30		
Hausman test		1		Prob ≥ 1.000		Prob ≥ 1.000			Prob ≥ 1.000		
		2		Prob ≥ 0.989		Prob ≥ 0.993			Prob ≥ 0.991		
		3		Prob ≥ 1.000		Prob ≥ 1.000			Prob ≥ 1.000		

Notes: (1) ***, **, and * denote a significance of 1%, 5%, and 10%, respectively; (2) Hausman test 1, H0: The CPMG estimator is preferred than CMG estimator; (3) Hausman test 2, H0: The CDFE estimator is preferred than CPMG estimator; (4) Hausman test 3, H0: The CDFE estimator is preferred than CMG estimator.

**Table 8:** Results of each aspect of financial development on CEL.

Aspect	FSZ	FST	FOP	FDP	FGR	FEF	FEC	Error Correction	Constant
Overall impact	0.369 *** (4.839)	−0.246 (−1.540)	0.133 ** (2.168)	0.221 *** (4.901)	−0.293 (−1.409)	−0.166 ** (−2.213)	−0.159 * (−1.798)	0.382 *** (3.829)	−0.224 *** (−4.338)
Eastern region	−0.496 *** (−3.439)	−0.410 (−0.021)	−0.284 ** (−2.267)	−0.142 ** (−2.199)	0.492 (1.503)	−0.398 *** (−4.839)	−0.451 *** (−4.223)	0.528 *** (5.490)	−0.308 *** (−4.113)
Middle region	0.313 *** (2.904)	0.351 *** (4.982)	0.192 *** (3.904)	0.129 *** (5.701)	0.929 (0.081)	0.042 (1.233)	0.148 *** (2.312)	0.551 *** (4.391)	−0.218 ** (−4.492)
Western region	0.235 ** (2.152)	−0.440 (−0.092)	0.926 (1.450)	0.105 * (−1.915)	−0.241 ** (−2.135)	0.282 (0.010)	−0.195 ** (−1.982)	0.345 *** (4.326)	−0.209 ** (−2.213)
Northeastern region	0.193 ** (1.998)	0.924 (0.241)	0.326 *** (6.132)	0.146 (0.021)	−2.410 (−0.199)	0.335 *** (4.213)	−0.153 (−1.288)	0.590 (1.095)	−0.037 *** (−5.192)

Notes: (1) ***, **, and * denote a significance of 1%, 5%, and 10%, respectively.

**Table 9:** Analysis of the impact of different aspects of financial development on carbon emissions.

Region	Impact	Reasons
**Financial Size** [99]		
Nationwide	+	1. Energy-intensive enterprises with small investment risk and high revenues use the extensive product mode. 2. Lack of government support and investment in energy conservation and emission reduction technologies. 3. Financial institutions do not focus on the design of low-carbon products.
Eastern region	−	1. The eastern region is a good location, and the financial size is in an early stage, so the costs of emission reduction are low. 2. The government guides carbon emissions positively, and the desire to reduce the emissions of companies in the eastern region is strong.
Middle region	+	
Western region	+	
North-eastern region	+	
**Financial openness** [100]		
Nationwide	+	1. The entry barrier for FDI in China is too low to ensure investment quality. The attraction for FDI is not sufficient, and the pursuit of FDI is blind. 2. The regulations are not perfect.
Eastern region	−	1. The eastern region can attract good foreign investment with high technology. 2. FDI in the western region is limited. 3. The reasons for middle and north-eastern regions are the same as those nationwide.
Middle region	+	
Western region	/	
North-eastern region	+	
**Financial depth** [101]		
Nationwide	+	1. The resources of financial credit agencies are directed to departments with high energy consumption. 2. FDI transforms industries with high energy consumption and pollution in China.
Eastern region	−	1. The technological advantage of the eastern region is obvious, and technology spill-over can offset the pollution. 2. In the eastern region, credit from financial institutions is placed in the green energy industry.
Middle region	+	
Western region	+	
North-eastern region	+	
**Financial efficiency** [102]		
Nationwide	−	1. Transformation rate of savings and deposits of financial institutions is high, and more financing can help change the product technology. 2. The financial environment for resident consumption improves, requiring low energy consumption products.
Eastern region	−	1. The eastern region has an obvious technological advantage with a high transformation rate of capital use. The situation in the north-eastern region is the opposite.
Middle region	/	
Western region	/	
North-eastern region	+	
**Financial ecology** [103]		
Nationwide	−	1. Technological innovation decreases carbon emissions. 2. Improvement in quality of life enhances awareness of energy conservation and emission reduction.
Eastern region	−	1. Insufficient control of technology innovation in the north-eastern region. 2. Awareness of carbon emissions reduction among residents is weak.
Middle region	−	
Western region	−	
North-eastern region	/	

Notes: (1) + indicates that the impact is positive; (2) − indicates that the impact is negative.

## Abbreviations

CEI	Carbon emission intensity
MG	Mean group
PMG	Pooled mean group
DFE	Dynamic fixed effect
FYP	Five-Year Plan
IDA	Index decomposing analysis
GMM	Generalized method of moment
LMDI	Logarithmic Mean Divisia Index
FE	Fixed effects
FGLS	Feasible generalized least squares
DK	Driscoll–Kraay standard errors
PCSE	Panel corrected standard errors
PLS	Partial least squares
SGPCA	Sequential global principal component analysis
FDIN	Financial development index
FDIS	Financial development index system
EWM	Entropy weight method
POP	Population
FIR	Financial interrelation ratio
UR	Urbanization
IS	Industrial structure
GDP	Gomestic gross products
TCE	Total carbon emission
CEI	Carbon emission intensity
CEPC	Carbon emission per capita
FSZ	Financial size
FST	Financial structure
FOP	Financial openness
FDP	Financial depth
FGR	Financial growth
FEF	Financial efficiency
FEC	Financial ecology
ARDL	Auto regressive distributed lag model
FD	Financial development
CEL	Carbon emission level
IPCC	Intergovernmental Panel on Climate Change
KMO	Kaiser-Meyer-Olkin
HE-HD	High emission-high development
HE-LD	High emission-low development
LE-HD	Low emission-high development
LE-LD	Low emission-low development
CD	Cross-sectional dependence

## Appendix A

**Table A1:** Contributions and policies of China related to the emissions reduction.

Time	Name of Policy	Related Contents
January 2017	The Comprehensive Working Plan for Energy Conservation and Emission Reduction during the 13th Five Year Plan	Assign total energy consumption and intensity goals for each province (city and district), propose goals for primary industries and clarify the control plan for CO_2_ total discharge levels in each region.
June 2016	Regulations of Industrial Energy Conservation	Emphasize a transaction system of energy use rights, clarify the management methods for energy saving and build a healthy and sound supervision system.
September 2014	Action Plan for Transformation and Updating of Energy Conservation and Emission Reduction of Coal (2014–2020)	Until 2020, the average coal consumption power supply of newly built coal-fired generating units should be lower than 300 g/kWh. Transformational active units should be lower than 310 g/kWh.
April 2014	Revise Environmental Protection Act	Highlight the cyclical utilization of resources and environmental improvement and protection to coordinate social and economic development and environmental protection.
September 2013	Action Plan for the Control of Air Pollution	Increase the selected ratio of raw coal to 70%.
July 2012	Revise Cleaner Production Promotion Law	Promote cleaner production, improve the utilization efficiency of resources, reduce and prevent pollution, and protect the environment.
August 2011	Comprehensive Working Plan for Energy Conservation and Emissions Reduction during the 12th Five Year Plan	Aim to decrease carbon emissions intensity by 17% in 2015 compared to 2010 within the national economy and social development plan of the 12th Five Year Plan.
April 2010	Revise Renewable Energy Law	Enhance the development and utilization of renewable energy and promote the rapid and orderly development of renewable energy industries.
April 2008	Energy Conservation Law	Promote energy conservation, improve the utilization efficiency of energy and promote comprehensive, harmonious and sustainable development.
March 2006	The 11th Five Year Plan	Aim to decrease energy consumption by 20%
August 2002	Kyoto Protocol	Aim to decrease carbon emissions by 40–45% by 2020 compared to 2005.

## Appendix B

**Table A2:** Integrated weight of each indicator.

Component	X1	X2	X3	X4	X5	X6	X7	X8	X9	X10	X11
Principal component method	0.0411	0.0330	0.0383	0.0362	0.0503	0.033	0.0682	0.072	0.0285	0.0355	0.0221
Entropy weight method	0.0398	0.0243	0.0456	0.0458	0.0494	0.0366	0.0485	0.0486	0.0398	0.0545	0.0486
Average	0.0405	0.0286	0.0419	0.0410	0.0498	0.0348	0.0583	0.0603	0.0342	0.0450	0.0354
Component	X_12_	X_13_	X_14_	X_15_	X_16_	X_17_	X_18_	X_19_	X_20_	X_21_	X_22_
Principal component method	0.0175	0.0321	0.0369	0.0691	0.0696	0.0653	0.0646	0.0245	0.0668	0.0268	0.0684
Entropy weight method	0.0444	0.0481	0.0470	0.0401	0.0485	0.0483	0.0493	0.0483	0.0497	0.0492	0.0457
Average	0.0309	0.0401	0.0419	0.0546	0.0590	0.0568	0.0569	0.0364	0.0582	0.0380	0.0570

## Appendix C. Cross-Sectional Dependence (CD), Panel Unit Root Test and Panel Cointegration Tests

Before discussing the empirical results of the relationship between financial development and carbon emissions, we need to solve three issues during the modelling process. One of the issues to be addressed is to test whether there is a cross-sectional dependence. Considering that the problem of inaccurate estimates of models can result from variable or residual correction across panel members owing to the spill-over effect and global common shocks, the cross-sectional dependence test is performed by applying the method developed by Pesaran [104]. The null hypothesis is that there is no cross-sectional dependence; accordingly, the alternative hypothesis is that cross-sectional dependence exists. The CD test is robust to the presence of multi-breaks in slope coefficients and in error variance. The results in Table A3 clearly show that the null hypothesis of no cross-sectional dependence is rejected in all models (*p*-value is 0.000).

**Table A3:** Cross-sectional dependence test.

Variable	CD-Test	*p*-Value	Abs(corr)	Variable	CD-Test	*p*-Value	Abs(corr)
CEPC	63.520	0.000	0.890	FST	52.350	0.000	0.775
GDPPC	75.430	0.000	0.976	FOP	53.880	0.000	0.719
IS	32.630	0.000	0.628	FDP	37.660	0.000	0.533
FD	53.970	0.000	0.692	FGR	48.700	0.000	0.626
FD^2^	48.400	0.000	0.627	FEF	38.170	0.000	0.515
FSZ	75.040	0.000	0.962	FEC	22.210	0.000	0.361

The second issue that needs to be addressed is to test for the presence of unit roots in panel data. Kim et al. [105] demonstrated that the PMG estimation of an ARDL regression could provide consistent estimators if there were a unique cointegration vector that defined the long-term relationship among variables regardless of the *I*(0) or *I*(1) process. Basically, this method does not require pre-unit root testing of the variables. PMG cannot be applicable to *I*(2) or a higher series. Consequently, we test the presence of a unit root by adopting the CIPS test proposed by Pesaran [106]. The null hypothesis of the test is that there is a unit root (non-stationarity) in the original time series; if the null is rejected in levels, the series is an *I*(0) process; if the null is not rejected in levels but is rejected in the first differences, the series is an *I*(1) process. The results in Table A4 indicate that the original level of the variables does not reject the null hypothesis that their series contain unit roots. However, the results for the estimation of the first difference of variables strongly reject the null hypothesis. Thus, the test proves that the series of all variables are *I*(1) processes. As a result, the variables need to be cointegrated into the model [107].

**Table A4:** Results of panel unit roots tests.

	Variables in Levels						Variables in First Differences					
	Constant w/o Trend			Constant w/Trend			Constant w/o Trend			Constant w/Trend		
No. lag	1	2	3	1	2	3	1	2	3	1	2	3
CEPC	0.796	0.883	0.891	0.996	0.994	0.998	0.000	0.000	0.000	0.000	0.000	0.000
GDPPC	0.574	0.629	0.735	0.965	0.978	0.993	0.000	0.000	0.000	0.000	0.000	0.000
IS	0.072	0.139	0.168	0.471	0.669	0.705	0.000	0.000	0.000	0.000	0.000	0.000
FD	0.905	0.945	0.911	0.920	0.946	0.998	0.000	0.000	0.000	0.000	0.000	0.000
FD^2^	0.918	0.936	0.900	0.972	0.963	0.977	0.000	0.000	0.000	0.000	0.000	0.000
FSZ	0.624	0.958	0.972	0.982	0.987	1.000	0.000	0.000	0.000	0.000	0.000	0.000
FST	0.946	0.998	0.999	0.703	0.996	0.999	0.000	0.000	0.000	0.000	0.000	0.000
FOP	0.874	0.889	0.903	0.641	0.993	0.817	0.000	0.000	0.000	0.000	0.000	0.000
FDP	0.318	0.856	0.774	0.621	1.000	0.999	0.000	0.000	0.000	0.000	0.000	0.000
FGR	0.568	0.626	0.888	0.825	0.945	0.991	0.000	0.000	0.000	0.000	0.000	0.000
FEF	0.838	0.851	0.998	0.617	1.000	0.995	0.000	0.000	0.000	0.000	0.000	0.000
FEC	0.302	0.492	0.517	0.806	0.885	0.793	0.000	0.000	0.000	0.000	0.000	0.000

Since most variables are non-stationary, the third issue to be solved is to test for panel cointegration, and the test results are shown in Table A5. The error correction-based cointegration tests developed by Westerlund [108] are used to test the null hypothesis of no cointegration between the carbon emissions and each explanatory variable. The first group of statistics, Gt and Gs, has a null hypothesis of no cointegration, and the alternative hypothesis is that there is at least one group of cointegration relationships. The second group of statistics, Pt and Ps, has the same null hypothesis as the first group, and the alternative hypothesis is that there is a cointegration relationship in the whole panel of data. From Table A5, it can be seen that most of the statistics of the error correction-based cointegration tests are significant, rejecting the null hypothesis at the 10% level or better.

**Table A5:** Results of panel cointegration tests.

CEPC	GDPPC	IS	FD	FD^2^	FSZ	FST	FOP	FDP	FGR	FEF	FEC
Gt	0.428 (0.006)	1.955 (0.975)	6.242 (1.000)	3.471 (1.000)	0.601 (0.026)	2.309 (0.000)	0.631 (0.736)	5.404 (0.000)	6.140 (1.000)	5.883 (0.000)	1.208 (0.006)
Ga	−3.716 (0.000)	−5.510 (0.000)	−2.657 (0.004)	−6.692 (0.000)	−5.091 (0.000)	−5.468 (0.000)	−5.667 (0.000)	−3.399 (0.000)	−2.307 (0.011)	−3.681 (0.000)	−5.379 (0.000)
Pt	0.815 (0.001)	−0.009 (0.096)	4.420 (1.000)	1.874 (0.070)	−0.776 (0.219)	−1.734 (0.042)	−2.589 (0.005)	1.628 (0.048)	4.616 (0.000)	3.517 (0.000)	0.676 (0.050)
Pa	−3.969 (0.000)	−5.407 (0.000)	−0.357 (0.010)	−3.214 (0.001)	−4.499 (0.000)	−7.197 (0.000)	−6.945 (0.000)	−2.991 (0.001)	−0.256 (0.009)	−2.805 (0.003)	−4.555 (0.000)

## Appendix D. Regional Division of China

**Table A6:** Regions division of China.

Region	Provinces
Eastern	Beijing, Tianjin, Hebei, Shanghai, Jiangsu, Zhejiang, Fujian, Shandong, Guangdong and Hainan.
Middle	Shanxi, Anhui, Jiangxi, Henan, Hubei and Hunan.
Western	Inner Mongolia, Guangxi, Chongqing, Sichuan, Guizhou, Yunnan, Xizang, Shaanxi, Gansu, Qinghai, Ningxia and Xinjiang.
Northeastern	Liaoning, Jilin and Heilongjiang.
